# Long-term efficacy of a Web-based computer-tailored nutrition education intervention for adults including cognitive and environmental feedback: a randomized controlled trial

**DOI:** 10.1186/s12889-015-1707-4

**Published:** 2015-04-12

**Authors:** Linda Springvloet, Lilian Lechner, Hein de Vries, Anke Oenema

**Affiliations:** Department of Health Promotion, School for Public Health and Primary Care (CAPHRI), Maastricht University, P.O. Box 616, Maastricht, MD 6200 the Netherlands; Faculty of Psychology and Educational Sciences, Open University of the Netherlands, P.O. Box 2960, Heerlen, DL 6401 the Netherlands

**Keywords:** Cognitive feedback, Environmental-level feedback, Self-regulation, Computer tailoring, Nutrition education, Fruit intake, Vegetable intake, Fat intake, High-energy snack intake

## Abstract

**Background:**

Unhealthy diets are prevalent in Western countries, especially among low-educated people. To have an effect on health, it is important that dietary changes are sustained over time. This study examines long-term effects of a cognitive and environmental-feedback version of a Web-based computer-tailored (CT) nutrition education intervention targeting fruit, vegetables, high-energy snacks and saturated fat.

**Methods:**

A randomized controlled trial was conducted with a basic (tailored intervention targeting individual cognitions and self-regulation processes; n = 456), plus (additionally targeting environmental-level factors; n = 459) and control group (generic nutrition information; n = 434). Participants were recruited from the general population and randomly assigned to a study group. Online self-reported questionnaires assessed fruit, vegetable, high-energy snack and saturated fat intake, self-regulation, self-control, and Body Mass Index (BMI) at baseline and nine-months post-intervention. Linear mixed model analyses examined group differences in change over time. Educational differences were examined by ‘group X time X education’ interaction terms. Effects were examined in the total sample and among participants who did not comply with dietary- or BMI guidelines.

**Results:**

The effects on vegetable intake in the total sample differed according to educational level (p = 02). Among low/moderate-educated participants, the basic version was significantly more effective in increasing vegetable intake than the control program (effect size (ES) = 0.32) and plus version (ES = 0.22). No effects were found for high-educated participants. Self-regulation change was significantly larger in the control group than in the basic (ES = 0.18) and plus (ES = 0.16) group.

**Conclusions:**

In general, both intervention versions did not result in long-term intervention effects. The exception was an effect of the basic version on self-reported vegetable intake among low/moderate-educated adults in the total sample. More research is needed on how targeting self-regulation processes and environmental-level factors in Web-based CT nutrition education interventions can increase long-term efficacy.

**Trial registration:**

Netherlands Trial Registry NTR3396.

**Electronic supplementary material:**

The online version of this article (doi:10.1186/s12889-015-1707-4) contains supplementary material, which is available to authorized users.

## Background

In most Western countries a low fruit and vegetable intake and a high intake of saturated fat and energy, such as from high-energy snacks, are highly prevalent [1-5], especially among lower educated people [5-9]. These unhealthy dietary intake patterns can have serious health consequences, such as obesity, cardiovascular diseases (CVD) and some forms of cancer [5,10]. It is therefore important to improve dietary intake patterns, also among lower educated people.

Improving dietary intake patterns can be achieved with computer-tailored (CT) nutrition education interventions, in which health information is adapted to individual needs and characteristics [11,12], and that can reach a large number of people at relatively low costs [13]. CT nutrition education interventions are shown to be effective in changing self-reported fruit, vegetable and fat intake in the short and medium term [13-16], also among lower educated people [17,18], but effect sizes (ES) are mostly small. To have an effect on health, it is important that dietary changes are sustained over time [19]. Although long-term efficacy is not often examined, some studies show positive long-term effects of CT interventions on self-reported fruit, vegetable and fat intake [13,16]. However, short- and medium-term effects are not always sustained and also in the long term ES are mostly small. Most CT nutrition education interventions mainly target individual cognitions, such as attitude and self-efficacy [16]. These determinants are an important first step in behavior change, because they can increase motivation, but they are not likely to result in sustained behavior change [20-22]. Innovating interventions by targeting additional determinants, such as self-regulation processes and environmental-level factors, may increase ES and long-term efficacy. Self-regulation is important to achieve sustained behavior change [23,24]. Self-regulation processes are associated with dietary behaviors [25] and targeting these processes in interventions has been shown to increase effects on dietary behaviors [26]. Targeting self-regulation processes in CT nutrition education interventions may therefore improve long-term efficacy [16]. Environmental-level factors may also be important drivers of (dietary) behaviors [27,28] and provide people with opportunities or barriers towards healthy eating [21,29]. Some environmental-level factors have been found to be associated with dietary behaviors. These are perceptions, such as perceived availability in the neighborhood [30,31] and the perception of price [7,30,32], and objective environmental-level factors, such as the home-availability [32-35]. In CT interventions it is possible to provide objective information and feedback on these environmental-level factors. However, in existing CT nutrition education interventions, environmental-level factors are only targeted to a limited extent and mostly in the form of perceived barriers that have to be overcome. Incorporating objective environmental-level information in Web-based CT nutrition education interventions is a novelty, but there is some evidence for long-term efficacy from the physical activity domain [36].

This study examines the long-term efficacy of two versions of a Web-based CT nutrition education intervention aimed at increasing fruit and vegetable intake and decreasing high-energy snack and saturated fat intake that incorporate these important elements [37]. The basic version targets individual cognitions (i.e. knowledge, awareness, attitude and self-efficacy) [38] and self-regulation processes (i.e. goal setting and action- and coping planning). The plus version additionally targets environmental-level factors (i.e. availability at home and perception of availability and price of food products in supermarkets). Both versions showed promising short- and medium-term effects on self-reported fruit, high-energy snack or fat intake, but not on vegetable intake, in both the total sample and among people who did not comply with the guidelines for one of the dietary outcomes [39]. For high-energy snack intake, indications for educational differences were found: the plus version was most effective for high-educated participants and the basic version was most effective for lower educated participants [39].

The aim of the present study was to examine the efficacy of both intervention versions at nine-months post-intervention on the self-reported intake of fruit, vegetables, high-energy snacks and saturated fat (i.e. primary outcomes) and on secondary outcomes, compared to generic nutrition information. Because improving the dietary intake patterns could eventually result in a decrease in Body Mass Index (BMI), BMI was included as a secondary long-term outcome measure. Self-control and self-regulation were also identified as secondary outcome measures, because changes in these skills could result in changes in behavior. The efficacy was evaluated in both the total study sample as well as among participants who did not comply with the guidelines for fruit, vegetables, high-energy snacks or fat or who were overweight (i.e. BMI > = 25 kg/m^2^) (i.e. risk groups). Another aim was to explore educational differences in long-term intervention effects. Additional, explorative, analyses were conducted to examine potential effects on compliance with dietary guidelines. Both versions were expected to be more effective than the generic nutrition information in changing primary and secondary outcomes.

## Methods

### Overview

A detailed description of the study protocol has been published elsewhere [37] (see Additional file 1) and therefore a summary of the methodology and protocol is described below. The trial is approved by the Medical Ethics Committee of the Erasmus Medical Centre in Rotterdam (NL35430.078.11/MEC-2010-408) and registered in the Dutch Trial Registry (NTR339).

### Study design

A three-group randomized controlled trial (RCT) was conducted from March 2012 to December 2013 in the Netherlands. Participants were randomly assigned to the basic intervention group (n = 456); the plus intervention group (n = 459); or the control group (n = 434). The outcome measures were assessed at baseline (T0) and nine-months post-intervention (T1). The whole study was conducted online.

### Study procedure

#### Participants

**Figure 1:**
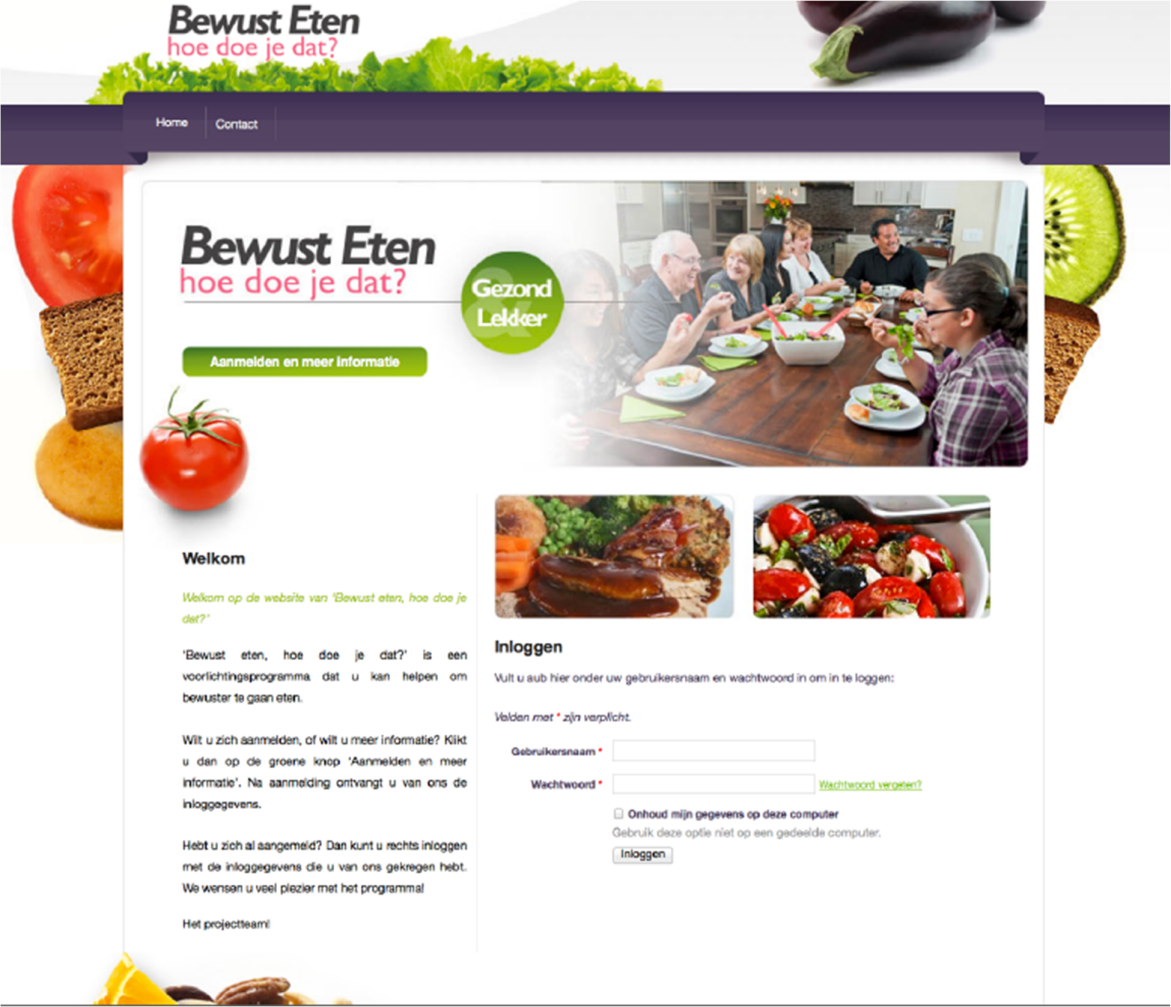
Screenshot of the study website.

The target group for this trial were adults (i.e. 20 to 65 years). A power calculation (power = 0.80; significance level α = .05) showed that 1,400 participants would be sufficient to detect a small intervention effect on all outcome measures (ES < 0.30) [37]. To account for dropout between each measurement, and a potential higher dropout among lower educated participants, 2,000 people needed to be recruited. Participants were recruited between March and October 2012 from the general population in five cities in the South of the Netherlands. The main recruitment strategy was sending personal mailings to 26,402 random home-addresses, which were obtained via municipalities. Additionally, Facebook advertisements, advertisements in (local) newspapers, local television and promotion activities in shopping malls (distribution of flyers and talking to people) were used for recruitment. People received a flyer with information about the goal, procedure and incentives for the study. Participants could sign up for the study by phone, e-mail or via the study website (Figure 1). Inclusion criteria were: being between 20 and 65 years of age, having a sufficient understanding of the Dutch language (in reading and writing) and having Internet access. Participants who were on a diet prescribed by a physician or dietician, had a medical condition that implies restrictions in eating behavior (e.g. CVD or bowel disease) or who were not willing to sign an informed consent were excluded from the study.

#### Procedure

**Figure 2:**
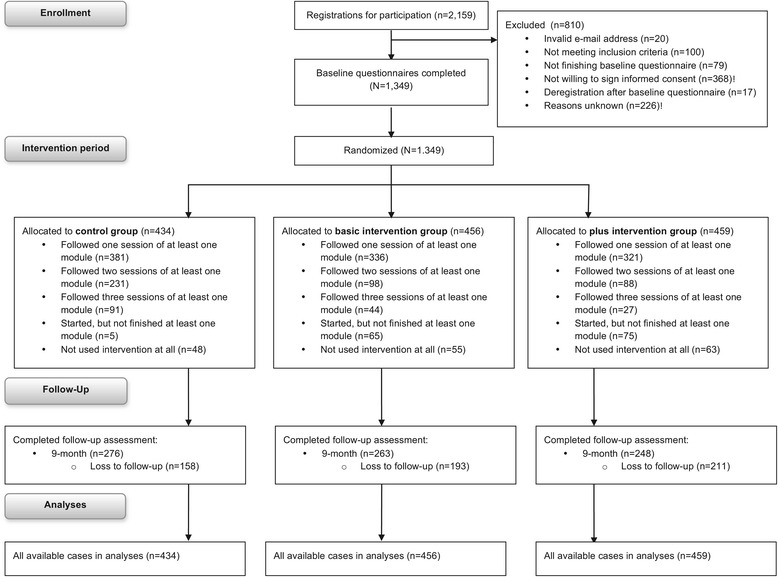
Overview of the procedure of the evaluation study and measurements.

After signing up for the study, a link to the online baseline questionnaire was sent via e-mail. One e-mail reminder for filling out the baseline questionnaire was sent two weeks after the initial invitation. The baseline questionnaire first assessed the inclusion- and exclusion criteria. People who met the inclusion criteria were asked to give online informed consent before they could continue with the baseline questionnaire. Additionally, a written informed consent form was sent via postal- or e-mail. Only people who signed and returned the written form were included in the study. One month after completing the baseline questionnaire, participants could start using the intervention. Randomization took place just before participants received the invitation to access the website, in order to conceal the allocation till the start of the intervention period. Within blocks of 10 participants, individual participants were randomly assigned to one of the study conditions in a computer-determined sequence, using the random number generation function of Microsoft Office Excel (Microsoft Corp, Redmond, WA, USA). Participants received a login code and password through e-mail. After logging in to the study website (Figure 1), participants were routed to the allocated intervention program (i.e. control, basic or plus). Participants were asked to visit the website at least three times during a two-month period. Nine months after the intervention period participants were asked by e-mail to fill out the follow-up questionnaire. E-mail reminders were sent two and four weeks after the initial invitation. Among participants who completed all four questionnaires of the trial, twenty iPad’s and 500 gift vouchers of 20 Euros were allotted. To improve the response, 20 extra gift vouchers were allotted for filling out the follow-up questionnaire. An overview of the study flow is shown in Figure 2.

### Intervention

The objective of the Web-based CT nutrition education intervention was to increase fruit and vegetable intake and decrease high-energy snack and saturated fat intake [37]. The two intervention versions were developed in a systematic way following the steps of the Intervention Mapping protocol [40] and were partly based on existing interventions [41,42]. Both versions consisted of four modules (i.e. fruit, vegetables, high-energy snacks and fat), each containing of three sessions that could be worked through during six consecutive weeks. Two weeks after each intervention visit, e-mail reminders were sent to prompt returning to the intervention, in order to evaluate progress toward achieving the behavioral goal or to receive feedback on another target behavior. Completion of the entire intervention took about 160 minutes. The first session took about 20 to 30 minutes to complete per module, and the second and third session about 10 to 20 minutes per module. The information was written at grade level 4–6 (i.e. years of education), in order to make the information comprehensible for lower educated people as well. The intervention was delivered via a website, on which participants could login (Figure 1). A pre-test among both high (n = 45) and lower educated (n = 20) people showed that both intervention versions were appreciated by the target group and that the information was usable and comprehensible, but there was also some room for improvement. Based on this pre-test, some small adaptations were made (e.g. decreasing the length of the text).

Both intervention versions were based on self-regulation theory [43], the Theory of Planned Behavior [38] and the Precaution Adoption Process Model [44] and targeted knowledge, awareness, intention, attitude, self-efficacy, goal setting and action- and coping planning. All four modules had a similar structure, except for the fat module that did not contain methods to target attitude and self-efficacy in the first session in order to limit participant burden, which was already high due to an extensive assessment questionnaire of fat intake. The three sessions were arranged according to the self-regulation phases pre-action, action and evaluation of the behavior change [23,45].

Participants could choose for which behavior(s) they wanted to receive feedback and guidance. After choosing a target behavior, the first session started with providing information to increase knowledge about the chosen behavior [46]. Subsequently, participants could monitor their behavior, based on which tailored personal, normative and comparative feedback was provided to increase awareness [40]. Attitude was targeted by providing feedback on self-selected advantages and disadvantages [40,47]. Feedback on self-selected potential barriers and difficult situations was provided to increase self-efficacy [40,46,47]. At the end of the first session participants could set a goal and formulate an implementation intention for when, where and how to make the behavior change using an if-then structure [40,46,48-50]. After the first session participants could start enacting their plans and initiate performing their new behavior for two weeks.

The second and third session provided the opportunity to evaluate the progress of the behavior change. Participants first monitored their goal-achievement in the past week and were provided with feedback on their progress [46,51,52]. When the goal had not been achieved, attitude and self-efficacy were targeted in order to stimulate participants to take a second attempt. All participants were stimulated to formulate coping plans for expected difficult situations [50]. If necessary, goals could be adapted, to make them more achievable or more challenging. The third session additionally provided information on how to maintain the behavior change, which was based on the three self-regulation phases [23,45] and described the different self-regulation steps participants could follow, for example: ‘what to do when your plan is not successful?’ or ‘what can you do when you relapse to your old habit?’.

#### Plus intervention version

The content of the plus version was identical to the basic version, but the first session additionally included environmental-level feedback on the availability and location of food products in the home food-environment and on the availability and prices of healthy food products in the supermarket the participant usually does his or her shopping. The second and third session were identical to the basic version. Because of the extra information in the plus version, the extra time to work through the first session of this version took approximately 5 to 10 minutes per module (i.e. 20 to 40 minutes extra for the whole intervention).

Before receiving feedback on dietary intake levels, participants could state for each target behavior at which supermarket they buy their food products (e.g. fruit). The tailored feedback that was provided contained the availability and price of products in this specific supermarket. The feedback on the availability and price of food products in the specific supermarket was incorporated in the feedback on attitude and self-efficacy. After selecting relevant disadvantages or barriers (e.g. ‘fruit is expensive’), participants received objective environmental-level information, which was presented as a list of selected food products that are available in the supermarket, with the price of the products if relevant for the disadvantage or barrier. This environmental-level feedback was also provided in a separate section. Before stating a goal and action plan, participants could review the list with the availability and prices of selected food products in their supermarket, relevant for the target behavior (e.g. in the module on fruit, only information about fruit was provided). Subsequently, participants could use this information to set goals and formulate action plans.

The availability and prices of selected food products were collected by observing participating supermarkets (n = 31) in the five cities in which the study was conducted. For supermarkets that did not provide permission for these observations (n = 27), more general information on availability and prices of this supermarket was provided. The information on availability was based on information that was available via websites or flyers of the supermarket, if possible (n = 13). When no information was available (n = 14), general information on availability of the selected food products (i.e. products that are available in most supermarkets) was provided to the participants. For prices, only general information (i.e. which products are usually least expensive in supermarkets) was provided for supermarkets that did not provide permission for observations.

In addition, the arrangement of the home food-environment was targeted. Participants could fill out whether they always have fruit, vegetables or high-energy snacks available at home and where they store fruit, vegetables or high-energy snacks. Subsequently, participants received feedback on possible improvements in availability and storage of products (e.g. ‘make sure you always have fruit available and store the fruit on a visible place, like in a fruit bowl’). Participants could use this information to create a more supportive home environment. This feedback consisted of approximately 8 to 10 lines of text. This section about the home food-environment was incorporated in the intervention before the objective information on availability and prices in supermarkets.

#### Control condition

The generic information for the control group also consisted of four modules, each consisting of three sessions that could be worked through in six consecutive weeks. Participants could choose for which behavior(s) they wanted to get information and received non-tailored information about fruit, vegetables, high-energy snacks and/or saturated fat, which was derived from general information that is available from the Netherlands Nutrition Centre [53] and the Dutch Vegetable and Fruit Centre [54]. Information was provided about, for example, the importance of complying with dietary guidelines, how individuals can eat more fruit and how individuals can maintain eating less saturated fat. The control program had the same name and layout as the intervention, was provided via the same website and similar reminders for (re-)visiting the program were sent.

### Measures

Online questionnaires were used to collect self-reported data on fruit, vegetable, high-energy snack and saturated fat intake, general self-regulation, self-control and BMI. The questionnaires additionally assessed behavior-specific determinants, but these are not included in this study.

#### Primary outcome measures

Vegetable and fruit intake were measured with a validated food frequency questionnaire (FFQ) [55,56]. Four items were used to measure vegetable intake in average grams per day. Participants were asked on how many days per week they usually consume cooked and raw vegetables or salads (ranging from 0–7 days per week) and how many tablespoons of cooked and raw vegetables or salads they usually eat on these days (ranging from one to six or more). One tablespoon of cooked vegetables represented 50 grams of vegetables and one tablespoon of raw vegetables or salads represented 25 grams of vegetables. Grams of vegetables per day were calculated by multiplying the frequency by the amount of tablespoons multiplied by grams, divided by 7 (days a week).

Six items were used to assess fruit intake in average amount of pieces of fruit per day. Participants were asked on how many days per week they usually consume citrus fruit, other fruit or (unsweetened) fruit juices (ranging from 0–7 days per week) and how many pieces or glasses they usually consume of citrus fruit, other fruit or fruit juices on these days (1 to seven or more). Amount of fruit consumed per day was calculated by multiplying the frequency by the amount of pieces or juices, divided by 7 (days a week).

Saturated fat intake was measured with a validated FFQ that assesses the frequency and quantity of a variety of food items eaten in the past week [57]. Participants were asked to report on how many days per week they usually consume a selection of food items during or between meals. If applicable, the quantity and kind of products (e.g. low-fat or full-fat milk) were also assessed. Based on this questionnaire, fat points were calculated, which represent grams of (saturated) fat. The total ‘fat score’ was based on 35 questions, assessing food products in the following categories: dairy products (n = 11), butter (n = 1), gravy (n = 3), sandwich fillings (n = 6), meat and cheese eaten at dinner (n = 4) and snacks (n = 10). Based on the frequency and amount of intake and the kind of product, fat points were assigned for each product group, ranging from 0 (lowest fat intake) to a maximum of 2 to 5 (highest fat intake, depending on how much fat a product group contains). The fat points for each product group were summed up to create a total fat points measure. In total, a maximum of 80 fat points could be obtained.

To measure snack intake, the questions on frequency of high-energy snack intake from the FFQ to measure fat intake [57] were used, in combination with extra items to measure the amount of snacks eaten per occasion. A total of 21 items measured high-energy snack intake, such as fried products, candy bars, cookies and chocolate. High-energy snack intake was calculated as the mean number of high-energy snacks eaten per day, by multiplying the frequency per week with the quantity, divided by 7 (days a week).

#### Secondary outcome measures

General self-regulation was measured using six items of the Self-Regulation Questionnaire [58]: ‘I have trouble to make plans that help me to reach my goal’ (reversed), ‘I have a hard time setting goals for myself’ (reversed), ‘When I have a goal, I can usually plan how to reach it’, ‘I give up quickly’ (reversed), ‘I set goals for myself and keep track of my progress’ and ‘when I try to change something, I pay attention on how I am doing’. Answers were on a 5-point scale (definitely not to definitely). Items were collapsed to a single variable by calculating the mean score over the six items (Cronbach’s alpha = 0.79).

Four items of the Brief Self Control measure [59] were used to measure self-control: ‘I am good at resisting temptation’, ‘ I think it is hard to change bad habits’ (reversed), ‘I refuse things that are bad for me’, ‘I wish I had more self-discipline’ (reversed). All answers ranged from definitely not to definitely on a 5-point scale. Items were collapsed to a single variable by calculating the mean score over the four items (Cronbach’s alpha = 0.77).

BMI was measured using self-reported height in meters at baseline and weight in kilograms (kg) at baseline and follow-up. Instructions on how to measure height and weight were provided to participants. BMI was calculated by dividing weight (kg) by the quadric of height in meters (m^2^).

#### Demographic factors

Sex (male vs. female), age (in years), place of residence (‘What is your place of residence?’: Heerlen, Roermond, Weert, Venlo, Venray), ethnicity and educational level were assessed in the baseline questionnaire. To assess educational level, participants had to indicate their highest attained educational level [60]. Educational level was first divided into three groups; high educated (higher vocational education and university), moderate educated (intermediate vocational education and higher secondary or pre-university education) and low educated (no education to lower general secondary education). Because differences in intake levels between low- and moderate-educated individuals are reported to be small [5], educational level was dichotomized into two groups; (0) high-educated and (1) low- and moderate-educated. Ethnicity (non-Western and Western) was defined according to the procedures of Statistics Netherlands [61]; participants were considered to be of Western ethnicity if both parents were born in Europe (except for Turkey), North America, Oceania, Indonesia or Japan. If at least one parent was born elsewhere, participants were considered to be of non-Western ethnicity.

### Statistical analyses

Multiple logistic regression analyses were conducted to test for selective dropout from the study and equality between the groups at baseline for demographics (i.e. gender, age, ethnicity, educational level, place of residence), study group and primary and secondary outcome measures at baseline.

Repeated measures analyses were conducted to study the intervention effects on the primary and secondary outcome measures. General linear mixed models with ‘time’ as a repeated statement and an unstructured covariance structure were used to study differences in changes over time between the three study groups (‘group X time’ interaction). Using a linear mixed model allowed for inclusion of cases with missing data, without replacement of missing values and therefore includes all randomized participants [62]. No clustering of observations of participants within cities was found, implying that including place of residence as an extra level was not indicated. In each analysis ‘time’, ‘group’ and a ‘group X time’ interaction were entered as independent variables and the ‘group X time’ interactions were interpreted. When the overall test statistic for the ‘group X time’ interaction was significant (p ≤ .05), in-depth results for group differences were examined and reported (i.e. unstandardized regression coefficients that represent the difference in change over time between two groups). Both intervention groups were compared to the control group and also to each other. An ES was calculated by dividing the unstandardized regression coefficient representing the difference in change over time between two groups with the square root of the variance. An ES below 0.50 was considered small, an ES between 0.50 and 0.80 moderate and an ES above 0.80 was considered large [63].

To examine potential educational differences in intervention effects, we explored for each outcome measure whether education moderated intervention effects, by adding a ‘group X time X education’ interaction term to the repeated measures analyses. If these interaction terms were statistically significant (p ≤ .05), stratified analyses were conducted and reported. The repeated measures analyses were conducted in both the total sample and the risk groups (i.e. participants who, at baseline, consumed less than 200 grams of vegetables, less than 2 pieces of fruit, more than 2 pieces of high-energy snacks, did not comply with gender- and age-specific guidelines for fat intake or were overweight).

In addition to the analyses on the main outcomes, effects on changes in compliance with the dietary guidelines were explored. Logistic mixed model analyses were conducted to examine the change in compliance with the dietary guideline from baseline to follow-up for each behavior separately (0 = not complying/1 = complying). In addition, a sum score for compliance with all dietary-specific guidelines (ranging from 0 to 4) was calculated, which was examined with linear mixed model analyses. The statistics of these analyses are not included in the tables.

Depending on the distribution of the outcome variable, the original or the log-transformed value was used in the repeated measures analyses. The repeated measures analyses were adjusted for place of residence and for demographic factors that differed between study groups or that were predictors for dropout. All tests were two-sided and alpha levels were set at .05. All analyses were performed with SPSS version 22.0 (IBM Corp, Armonk, NY, USA).

## Results

### Background characteristics

A total of 1,349 participants were included in the analyses. Participants were on average 49.05 years (SD = 10.62), 64.6% were female, 1.3% had a non-Western ethnic background and 54.3% had a low/moderate educational level (Table 1). Participants consumed on average 159.12 grams (SD = 69.24) of vegetables, 1.85 pieces (SD = 1.29) of fruit, 3.34 pieces (SD = 2.98) of high-energy snacks and 17.91 ‘fat-points’ (SD = 6.07) a day. Participants scored on average 3.48 (SD = 0.79) on self-regulation and 2.96 (SD = 0.86) on self-control. The mean BMI was 25.64 kg/m^2^ (SD = 4.20). On average, participants complied with 1.59 (SD = 1.12) of the 4 dietary guidelines at baseline. 1,014 participants (75.2%) did not comply with the recommendation of 200 grams of vegetables a day, 803 participants (59.5%) consumed less than 2 pieces of fruit per day, 808 participants (59.9%) consumed more than 2 high-energy snacks per day, 627 participants (46.5%) had a higher fat intake than recommended and 682 participants (50.6%) had a BMI above 25 kg/m^2^. Participants in the basic (p = .02) and plus (p = .02) groups were younger than participants in the control group. No other differences between groups were found.

**Table 1:** **Participant characteristics at baseline**

			**Total (N = 1349)**	**Control (n = 434)**	**Basic (n = 456)**	**Plus (n = 459)**	**OR [95% CI] for group differences** ^**a**^		
							**Basic vs. control**	**Plus vs. control**	**Plus vs. basic**
**Background characteristics**									
	**Age (years), mean (SD)**		49.05 (10.62)	50.01 (10.40)	48.63 (11.10)	48.54 (10.30)	0.98 [0.97,0.997]^*^	0.98 [0.97,0.996]^*^	1.00 [0.99, 1.01]
	**Gender, n (%)**								
		Male	478 (35.4)	145 (33.4)	165 (36.2)	168 (36.6)	1	1	1
		Female	871 (64.6)	289 (66.6)	291 (63.8)	291 (63.4)	0.83 [0.61, 1.11]	0.81 [0.60, 1.10]	1.01 [0.75, 1.35]
	**Ethnicity (n = 1348), n (%)**								
		Western	1,330 (98.7)	425 (98.2)	451 (98.9)	454 (98.9)	1	1	1
		Non-western	18 (1.3)	8 (1.8)	5 (1.1)	5 (1.1)	0.48 [0.15, 1.52]	0.44 [0.14, 1.38]	0.90 [0.25, 3.20]
	**Educational level, n (%)**								
		High	616 (45.7)	184 (42.4)	232 (50.9)	200 (43.6)	1	1	1
		Low/moderate	733 (54.3)	250 (57.6)	224 (49.1)	259 (56.4)	0.78 [0.59, 1.04]	1.001 [0.76, 1.33]	1.29 [0.98, 1.69]
	**Place of residence (i.e. cities in the Netherlands), n (%)**								
		Heerlen	323 (23.9)	103 (23.7)	113 (24.8)	107 (23.3)	1	1	1
		Roermond	217 (16.1)	69 (15.9)	78 (17.1)	70 (15.3)	1.04 [0.68, 1.61]	1.00 [0.65, 1.54]	0.97 [0.64, 1.48]
		Weert	251 (18.6)	77 (17.7)	82 (18.0)	92 (20.0)	1.04 [0.68, 1.58]	1.19 [0.79, 1.81]	1.18 [0.79, 1.78]
		Venlo	304 (22.5)	104 (24.0)	93 (20.4)	107 (23.3)	0.82 [0.55, 1.22]	1.02 [0.69, 1.50]	1.27 [0.85, 1.88]
		Venray	254 (18.8)	81 (18.7)	90 (19.7)	83 (18.1)	1.06 [0.70, 1.61]	1.05 [0.69, 1.59]	1.00 [0.67, 1.51]
**Primary outcome measures**									
	**Vegetable intake (grams)**								
		Mean (SD)	159.12 (69.24)	157.73 (64.54)	162.68 (72.76)	156.91 (69.94)	1.00 [0.999,1.003]	1.00 [0.998,1.003]	1.00 [0.997,1.002]
		Not complying, n (%)	1014 (75.2)	330 (76.0)	338 (74.1)	346 (75.4)			
	**Fruit intake (pieces)**								
		Mean (SD)	1.85 (1.29)	1.80 (1.23)	1.92 (1.36)	1.81 (1.27)	1.11 [0.98, 1.26]	1.06 [0.93, 1.20]	0.94 [0.83, 1.06]
		Not complying, n (%)	803 (59.5)	261 (60.1)	263 (57.7)	279 (60.8)			
	**High-energy snack intake (pieces)**								
		Mean (SD)	3.34 (2.98)	3.19 (2.74)	3.30 (2.94)	3.51 (3.24)	1.04 [0.97, 1.10]	1.05 [0.99, 1.11]	1.02 [0.96, 1.08]
		Not complying, n (%)	808 (59.9)	251 (57.8)	275 (60.3)	282 (61.4)			
	**Fat intake (‘Fat-points’) (n = 1348)**								
		Mean (SD)	17.91 (6.07)	17.99 (6.07)	17.60 (6.09)	18.13 (6.05)	0.97 [0.94, 1.003]	0.98 [0.95, 1.01]	1.01 [0.98, 1.04]
		Not complying, n (%)	627 (46.5)	197 (45.4)	203 (44.5)	227 (49.5)			
	**Total number of guidelines complying with, mean (SD)**		1.59 (1.12)	1.61 (1.12)	1.63 (1.11)	1.53 (1.12)	0.84 [0.68, 1.03]	0.88 [0.71, 1.07]	1.04 [0.84, 1.28]
**Secondary outcome measures (n = 1347)**									
	**Self-regulation, mean (SD)**		3.48 (0.79)	3.44 (0.80)	3.55 (0.78)	3.45 (0.78)	1.15 [0.93, 1.43]	0.98 [0.79, 1.22]	0.88 [0.71 1.08]
	**Self-control, mean (SD)**		2.96 (0.86)	2.91 (0.88)	3.02 (0.84)	2.96 (0.87)	1.13 [0.91, 1.39]	1.21 [0.98, 1.49]	1.06 [0.86, 1.31]
	**BMI (kg/m** ^**2**^ **)**								
		Mean (SD)	25.64 (4.20)	25.72 (4.40)	25.43 (4.06)	25.76 (4.14)	1.01 [0.97, 1.04]	1.02 [0.98, 1.05]	1.01 [0.98, 1.05]
		> = 25 kg/m2, n (%)	682 (50.60)	220 (50.9)	222 (48.7)	240 (52.3)			

^a^Logistic regression model with age, gender, ethnicity, educational level, place of residence, fruit intake, vegetable intake, high-energy snack intake, fat intake, number of guidelines complying with, self-regulation, self-control and BMI as independent variables; ^*^Significant at p ≤ .05.

### Dropout

The baseline questionnaire was filled out by 1,349 participants, of which 787 filled out the complete follow-up questionnaire (41.7% dropout) (Figure 2). Dropout was higher among younger compared to older participants (OR = 1.02, 95% CI [1.002, 1.004], p < .001) and among low/moderate-educated compared to high-educated participants (OR = 1.33, 95% CI [1.05, 1.68], p = .02). Dropout in the plus group was higher than in the control group (OR = 1.45, 95% CI [1.10, 1.91], p = .01). A higher score on self-control was associated with a lower dropout rate (OR = 0.82, 95% CI [0.68, 0.97], p = .02).

### Primary outcome measures

#### Dietary intake

Fruit intake increased over time (p < .001), but no differences were found between the groups (p = .50) (Tables 2 and 3). Similar results were found in the risk group (Tables 2 and 3).

**Table 2:** **Estimated marginal means for primary outcome measures (n = 1,349)** ^**a**^

			**Control**			**Basic**			**Plus**		
			**Baseline, mean (SE)**	**Follow-up, mean (SE)**	**Difference [95% CI]**	**Baseline, mean (SE)**	**Follow-up, mean (SE)**	**Difference [95% CI]**	**Baseline, mean (SE)**	**Follow-up, mean (SE)**	**Difference [95% CI]**
**Fruit (pieces)**											
	*Total sample*		1.81	1.88	0.07	1.94	2.10	0.16	1.82	1.99	0.16
	(n = 1349)		(0.06)	(0.07)	[−0.05, 0.19]	(0.06)	(0.07)	[0.04, 0.28]^*^	(0.06)	(0.07)	[0.04, 0.29]^*^
	*Risk group*		1.04	1.41	0.37	1.04	1.51	0.47	1.03	1.56	0.53
	(n = 803)		(0.03)	(0.06)	[0.25, 0.49]^*^	(0.03)	(0.07)	[0.35, 0.59]^*^	(0.03)	(0.07)	[0.41, 0.66]^*^
**Vegetables (grams)**											
	*Total sample*										
		High-educated	162.97	165.51	2.53	174.40	177.31	2.90	165.61	180.28	14.68
		(n = 616)	(5.09)	(6.38)	[−9.13,14.19]	(4.49)	(5.64)	[−7.48, 13.29]	(4.85)	(6.30)	[3.04, 26.31]^*^
		Low/moderate-educated	152.84	148.90	−3.93	151.59	169.75	18.16	151.17	154.47	3.29
		(n = 733)	(4.39)	(4.98)	[−12.35, 4.48]	(4.63)	(5.62)	[8.45, 27.87]^*^	(4.31)	(5.28)	[−5.84, 12.42]
	*Risk group*										
		High-educated	135.30	152.46	17.15	132.93	146.22	13.28	133.47	161.19	27.72
		(n = 438)	(3.31)	(6.24)	[4.95, 29.36]^*^	(3.04)	(5.84)	[1.81, 24.75]^*^	(3.20)	(6.39)	[15.19, 14.26]^*^
		Low/moderate-educated	124.70	135.84	11.13	124.41	150.10	25.69	120.75	132.67	11.92
		(n = 576)	(2.99)	(4.83)	[2.34, 19.93]^*^	(3.09)	(5.43)	[15.66, 35.71]^*^	(2.92)	(5.11)	[2.48, 21.36]^*^
**Snacks (pieces)** ^**b**^											
	*Total sample*		3.22	2.68	−0.53	3.29	2.40	−0.89	3.50	2.49	−1.01
	(n = 1349)		(0.14)	(0.13)	[−0.79, −0.28]^*^	(0.14)	(0.13)	[−1.15, −0.64]^*^	(0.14)	(0.13)	[−1.28, −0.75]^*^
	*Risk group*		4.75	3.66	−1.09	4.77	3.12	−1.64	5.03	3.18	−1.85
	(n = 808)		(0.19)	(0.19)	[−1.48, −0.69]^*^	(0.18)	(0.19)	[−2.04, −1.25]^*^	(0.18)	(0.19)	[−2.25, −1.45]^*^
**Fat (‘fat-points’)**											
	*Total sample*		17.95	16.76	−1.19	17.60	15.83	−1.77	18.08	16.15	−1.93
	(n = 1349)		(0.29)	(0.32)	[−1.72, −0.66]^*^	(0.29)	(0.32)	[−2.31,-1.24]^*^	(0.29)	(0.33)	[−2.48, −1.38]^*^
	*Risk group*		22.98	19.97	−3.02	22.65	19.29	−3.37	22.82	19.21	−3.61
	(n = 627)		(0.30)	(0.45)	[−3.82, −2.22]^*^	(0.30)	(0.45)	[−4.18, −2.55]^*^	(0.28)	(0.45)	[−4.43, −2.78]^*^

^a^Based on linear mixed model including place of residence, age, education, study group, time, ‘group X time’; ^b^Significance tests based on natural logarithm of high-energy snacks; ^*^Significant at p ≤ .05.

**Table 3:** **Results general linear mixed models for primary outcome measures**

							**P-value**	**B [95% CI]** ^**a**^	**ES** ^**b**^
**Fruit intake**									
	Type III tests total sample (n = 1349)								
		Group x time x education^c^					.52	–	
		Group x time^d^					.50	–	
		Time^e^					<.001^*^	–	
	Type III tests risk group (n = 803)								
		Group x time x education^c^					.24	–	
		Group x time^d^					.17	–	
		Time^e^					<.001^*^	–	
**Vegetable intake**									
	Type III tests total sample (n = 1349)								
		Group x time x education^c^					.02^*^	–	
			*Low/moderate-educated participants (n = 733)*						
				Group x time^d^			.003^*^	–	
				Time^e^			.07	–	
					Differences over time between groups for low/moderate-educated participants				
						Basic vs. control^d^	.001^*^	22.09 [9.25, 34.93]	0.32
						Plus vs. control^d^	.25	7.23 [−5.19, 19.64]	0.11
						Plus vs. basic^d^	.03^*^	−14.87 [−28.20, −1.54]	0.22
			*High-educated participants (n = 616)*						
				Group x time^d^			.25	–	
				Time^e^			.052	–	
	Type III tests risk group (n = 1014)								
		Group x time x education^c^					.03^*^	–	
			*Low/moderate-educated participants (n = 576)*						
				Group x time^d^			.07	–	
				Time^e^			<.001^*^	–	
			*High-educated participants (n = 438)*						
				Group x time^d^			.23	–	
				Time^e^			<.001^*^	–	
**High-energy snack intake** ^**f**^									
	Type III tests total sample (n = 1349)								
		Group x time x education^c^					.71	–	
		Group x time^d^					.14	–	
		Time^e^					<.001^*^	–	
	Type III tests risk group (n = 808)								
		Group x time x education^c^					73	–	
		Group x time^d^					.13	–	
		Time^e^					<.001^*^	–	
**Fat intake**									
	Type III tests total sample (n = 1349)								
		Group x time x education^c^					.82	–	
		Group x time^d^					.13	–	
		Time^e^					<.001^*^	–	
	Type III tests risk group (n = 627)								
		Group x time x education^c^					.71	–	
		Group x time^d^					.60	–	
		Time^e^					<.001^*^	–	

^a^B = Unstandardized regression coefficient for difference between groups in change over time; ^b^ES = Effect size; ^c^Based on linear mixed model including place of residence, age, education, study group, time, ‘group X time’, ‘time X education’, ‘group X education’ and ‘group X time X education’; ^d^Based on linear mixed model including place of residence, age, education, study group, time, ‘group X time’; ^e^ Based on linear mixed model including place of residence, age, education, study group, time; ^f^Repeated measures analyses on natural logarithm of high-energy snacks, estimates based on original variable; ^*^Significant at p ≤ .05.

For vegetable intake, the interaction of ‘group X time X education’ was significant in both the total sample (p = .02) and risk group (p = .03) (Table 3). Among low/moderate-educated participants in the total sample the basic version resulted in a larger increase in vegetable intake compared to the control program (ES = 0.32, p = .001) and the plus version (ES = 0.22, p = .03) (Tables 2 and 3). Among high-educated participants in the total sample, the change over time in vegetable intake was marginally significant (p = .052) and no group differences were found (p = .25). In the risk group no group differences were found among both high-educated (p = .23) and low/moderate-educated participants (p = .07) (Tables 2 and 3).

Snack intake decreased in the total sample (p < .001), but the change over time did not differ between the groups (p = .14) (Tables 2 and 3). The same pattern was found in the risk group (Tables 2 and 3).

The intake of saturated fat was lower at follow-up than at baseline (p < .001), but this change over time was not different between the groups (p = .13) (Tables 2 and 3). In the risk group similar results were found (Tables 2 and 3).

#### Compliance with dietary guidelines

At baseline 40.5, 24.8, 40.1 and 53.5% of the participants complied with the guidelines for fruit, vegetables, high-energy snacks and fat respectively. At follow-up, the percentages were 49.8, 28.7, 55.2 and 63.3 respectively. Participants complied with 1.59 (SD = 1.12) of the 4 dietary guidelines at baseline and 1.97 (SD = 1.11) guidelines at follow-up. No intervention effects on compliance with guidelines were found for fruit (p = .65), vegetables (p = .27) and snacks (p = .58). For compliance with the age- and gender-specific guidelines for fat intake group differences were found (p = .04): the plus version had greater effect than the control program (B = 0.42, ES = 0.42, p = .02). No difference between the basic and control group was found (B = 0.33, ES = 0.33, p = .06). The change over time in the total number of dietary guidelines participants comply with differed between the study groups (p = .02) and was larger in both the basic (B = 0.22, ES = 0.20, p = .02) and plus group (B = 0.22, ES = 0.20, p = .02) than in the control group.

### Secondary outcome measures

The results for the secondary outcome measures are shown in Tables 4 and 5. Differences in change over time between the three groups were found for self-regulation (p = .04), with a lower increase in self-regulation in both the basic (ES = −0.18, p = .02) and plus group (ES = −0.16, p = .04) compared to the control group. Self-control increased over time (p < .001), but no differences between the three groups were found (p = .29). There was a slight decrease in BMI over time in the total sample (p = .001), but this change was not different between the groups (p = .17). Among overweight participants BMI also decreased over time (p < .001), but no group differences were found (p = .30).

**Table 4:** **Estimated marginal means for secondary outcome measures** ^**a**^

	**Control**			**Basic**			**Plus**		
	**Baseline, mean (SE)**	**Follow-up, mean (SE)**	**Difference [95% CI]**	**Baseline, mean (SE)**	**Follow-up, mean (SE)**	**Difference [95% CI]**	**Baseline, mean (SE)**	**Follow-up, mean (SE)**	**Difference [95% CI]**
**Self-regulation**	3.45	3.66	0.21	3.56	3.61	0.05	3.48	3.55	0.07
**(n = 1348)** ^**b**^	(0.04)	(0.05)	[0.12, 0.30]^*^	(0.04)	(0.05)	[−0.04, 0.15]	(0.04)	(0.05)	[−0.03, 0.17]
**Self-control**	2.91	3.17	0.26	3.02	3.19	0.17	2.98	3.22	0.24
**(n = 1348)** ^**c**^	(0.04)	(0.05)	[0.17, 0.34]^*^	(0.04)	(0.05)	[0.08, 0.25]^*^	(0.04)	(0.05)	[0.15, 0.33]^*^
**BMI (kg/m** ^**2**^ **)**									
*Total sample*	25.61	25.61	0.00	25.45	25.32	−0.12	25.73	25.56	−0.17
(n = 1347)	(0.20)	(0.20)	[−0.13, 0.13]	(0.19)	(0.20)	[−0.26, 0.01]	(0.19)	(0.20)	[−0.31, −0.04]^*^
*Risk group*	28.78	28.66	−0.12	28.54	28.23	−0.22	28.64	28.26	−0.38
(n = 682)	(0.24)	(0.26)	[−0.35, 0.10]	(0.24)	(0.26)	[−0.45, −0.001]^*^	(0.23)	(0.25)	[−0.61, −0.14]^*^

^a^Based on linear mixed model including place of residence, age, education, study group, time, ‘group X time’; ^b^Measured on a 5-point scale; ^c^Measured on a 4-point scale; ^*^Significant at p ≤ .05.

**Table 5:** **Results general linear mixed models for secondary outcome measures**

					**P-value**	**B [95% CI]** ^**a**^	**ES** ^**b**^
**Self-regulation**							
	Type III tests total sample (n = 1348)						
		Group x time x education^c^			.88	–	–
		Group x time^d^			.04^*^	–	–
		Time^e^			<.001^*^	–	–
			Differences over time between groups				
				Basic vs. control^d^	.02^*^	−0.16 [−0.29, −0.02]	−0.18
				Plus vs. control^d^	.04^*^	−0.14 [−0.17, −0.004]	−0.16
				Plus vs. basic^d^	.81	0.02 [−0.12, 0.15]	0.02
**Self-control**							
	Type III tests total sample (n = 1348)						
		Group x time x education^c^			.29	–	–
		Group x time^d^			.29	–	–
		Time^e^			<.001*	–	–
**BMI**							
	Type III tests total sample (n = 1347)						
		Group x time x education^c^			.22	–	–
		Group x time^d^			.17	–	–
		Time^e^			.01^*^	–	–
	Type III tests risk group (n = 682)						
		Group x time x education^c^			.64	–	–
		Group x time^d^			.30	–	–
		Time^e^			<.001^*^	–	–

^a^B = Unstandardized regression coefficient for difference between groups in change over time; ^b^ES = Effect size; ^c^Based on linear mixed model including place of residence, age, education, study group, time, ‘group X time’, ‘time X education’, ‘group X education’ and ‘group X time X education’; ^d^Based on linear mixed model including place of residence, age, education, study group, time, ‘group X time’; ^e^ Based on linear mixed model including place of residence, age, education, study group, time; ^*^Significant at p ≤ .05.

## Discussion

This study examined the long-term effects and educational differences in effects of a basic version (targeting individual cognitions and self-regulation processes) and plus version (additionally targeting environmental-level factors) of a Web-based CT nutrition education intervention on self-reported intake of fruit, vegetables, high-energy snacks and saturated fat and self-regulation, self-control and BMI compared to generic nutrition information. The only significant intervention effect was found for self-reported vegetable intake in the total sample: among low/moderate-educated participants the basic version was more effective compared to both the control program and plus version. Self-regulation slightly increased in the control group only. No intervention-effects were found for fruit, high-energy snack and fat intake, self-control and BMI. The exploratory analyses showed that the plus version had greater effect on compliance with age- and gender-specific guidelines for fat intake compared to the control group. Both the basic and plus version were more effective than the control program in increasing the total number of dietary guidelines participants comply with.

The long-term effect of the basic version on self-reported vegetable intake is promising and in line with some other Web-based CT nutrition education interventions [13,16]. The effect was only found for low/moderate-educated participants, which is promising, because this is an important target group for nutrition education. Based on the results of the short- and medium-term effect evaluation, we expected the least effect on vegetable intake compared to the other dietary behaviors, because no intervention effects were found on vegetable intake earlier [39]. This may suggest a sleeper effect. However, no effects were found among people with a lower vegetable intake than recommended, which may indicate that already healthy behaviors were further increased. This is positive, but not the primarily intended effect.

By including self-regulation processes and multiple tailored feedback moments in the intervention it was expected to achieve long-term effects, because by following the steps of the self-regulation process, people can (self-)regulate their dietary behavior and learn to adapt their behavior when needed in order to achieve self-set goals [43]. Because people learn to react on changes in their environment or actions, self-regulation of behavior can result in sustained behavior change [24,50,64]. However, the short- and medium-term intervention effects on fruit, high-energy snack and fat intake that were found earlier [39] were not sustained in the long term. These findings are in line with those of previous studies, because although some previous Web-based CT interventions show promising long-term effects on the intake of fruit, vegetables and fat, not finding long-term effects is often reported [13,16]. Another Web-based CT self-regulation intervention, aimed at weight management for overweight adults, did also not show effects on dietary intake (saturated fat and snack intake), but the effects of that intervention were examined at one and six months post-intervention only [65]. There are several factors that may explain why we did not find long-term effects. First of all, the ES that were found at the short and medium term were already small, which may make it very likely that these effects faded out over time. Another explanation may be that the self-regulation tools were not optimally incorporated and used. For example, the progression and maintenance of the behavior change were targeted in the second and third session of the intervention. However, exposure to these two follow-up sessions was very low, which is often observed in online interventions [66,67]. Consequently, participants may have not sufficiently integrated self-regulation skills in daily life, which may have resulted in not adopting new habits. This may also explain why we did not find long-term effects on self-regulation skills. Because self-regulation is important for sustained behavior change, and Web-based interventions have many advantages, more research is needed on how self-regulation tools and techniques can be effectively incorporated in Web-based CT interventions and how prolonged exposure and use of such interventions can be increased.

It was expected to achieve long-term effects of the plus version by inducing changes in the home food-environment and in the perception of availability and price of food products in the supermarkets, at least for fruit and high-energy snack intake for which short- and medium-term effects were found [39]. Not finding long-term effects may indicate that the environmental-level factors that were targeted have not been (sufficiently) changed by the intervention. One explanation may be that the environmental-level feedback was not extensive enough to achieve sustained changes. The home food-environment, for example, was only a small part of the intervention, while adapting the home food-environment is a complex behavior that includes multiple behavioral determinants, such as awareness and self-efficacy. The literature shows that environmental-level factors are important in changing dietary behaviors [7,27,28,30-35], but the way these factors were targeted in this intervention was not effective in achieving long-term effects on dietary behavior. No previous Web-based CT nutrition education interventions that provide objective environmental-level information are known. However, for physical activity there are promising long-term effects of providing objective environmental-level information among older adults [68,69]. Targeting environmental-level factors is probably more complex for dietary behaviors than for physical activity, because the objective information is more sensitive to fluctuations and there are a large variety of foods available within each food group. Therefore, more research is needed on how these factors can be targeted in order to achieve long-term effects. Future studies could, for example, examine whether more extensively targeting environmental-level factors increases long-term efficacy and sustained behavior change.

Although no effects on self-reported saturated fat intake were found, the plus version did increase the number of participants who comply with the guidelines. In addition, both intervention versions were more effective in increasing the total number of dietary guidelines participants comply with. This is a promising result, because complying with dietary guidelines is important for decreasing health risks [5,10]. However, this effect may have been caused by a small increase in intake among participants who already almost complied with the guidelines, so the actual effect may be of limited importance. In addition, this was not the outcome that this study was initially aimed at.

Not finding intervention effects on BMI is in line with another CT self-regulation intervention [65]. An effect on BMI could be expected when substantial changes in obesity-related behaviors are initiated and maintained over time. Both intervention versions appeared not to result in long-term changes in dietary behaviors and did, consequently, not result in a change in BMI. In order to influence important health outcomes, such as BMI, it is important to examine how intervention effects can be increased and sustained.

The effect of the control program on self-regulation was unexpected, because the control program only provided some general information on self-regulation processes (e.g. ‘making plans can be useful’). In the assessment questionnaires, self-regulation was mainly measured by items about planning, monitoring and goal setting. Participants in the intervention groups were guided through these processes of self-regulation and may have been more aware of what planning, monitoring and goal setting actually consist of. Therefore, they may have experienced it to be more difficult than expected, resulting in being more critical in answering these questions, which may explain the smaller increase in self-regulation in both intervention groups. In addition, self-regulation was measured as general self-regulation, instead of behavior specific self-regulation. This measure may have been too general to detect changes on self-regulation related to the specific dietary behaviors, which could decrease potential intervention effects on (behavior specific) self-regulation.

### Limitations

Some limitations of this study should be taken into account when interpreting the results. Firstly, a selective sample of the population may have been recruited due to selective response. Intake levels were more favorable compared to the general Dutch population [5], which may indicate that the study population was more motivated for or interested in healthy nutrition. In addition, dropout from the study was high and was selective for age, intervention group and self-control. A high dropout is often reported in other Web-based CT interventions [65,67,70,71], but may influence the intervention effects. By conducting linear mixed model analyses and by correcting the analyses for predictors for dropout, an attempt to minimize bias potentially caused by selective dropout was made. The selective sample and high and selective dropout may have decreased the external validity of the results. Therefore, the results are only generalizable to Dutch adults who are interested in healthy eating. Secondly, although mostly validated questionnaires were used to measure dietary intake, the study relied on self-reported data. This may be less valid than using more objective instruments, such as biomarkers, and may have resulted in, for example, an overestimation of the intervention effects [72]. The questionnaires are, however, suitable to rank people according to their intake levels and according to changes and differences in intake levels [56,57]. Thirdly, the items to measure high-energy snack intake were derived from validated questionnaires and used in previous studies (e.g. [65,73]), but validity and reliability of these items to measure the amount of snacks eaten per day are not known and these results should therefore be interpreted with caution. Lastly, self-control and self-regulation were measured with a small number of items derived from validated questionnaires, but validity of using this set of items only is not known.

## Conclusion

In general, both intervention versions did not result in long-term intervention effects. The exception was an effect of the basic version on self-reported vegetable intake among low/moderate-educated adults in the total sample. Because of the potential importance of self-regulation and environmental-level factors in dietary behavior change, more research is needed on how targeting these factors in Web-based CT nutrition education interventions can increase long-term efficacy.

## Additional file

Additional file 1:
**Study protocol.**

## Abbreviations

BMI: Body mass index
CI: Confidence interval
CT: Computer-tailored
CVD: Cardiovascular diseases
ES: Effect size(s)
FFQ: Food frequency questionnaire
SE: Standard error
