# Supplementary material for: Long-term efficacy of a Web-based computer-tailored nutrition education intervention for adults including cognitive and environmental feedback: a randomized controlled trial
Source: BMC Public Health. 2015 Apr 12;15:372. doi: 10.1186/s12889-015-1707-4 (PMC4424509; doi:10.1186/s12889-015-1707-4)
Supplement: Additional file 1: — Study protocol. [file 12889_2015_1707_MOESM1_ESM.pdf]

BMC Public Health

STUDY PROTOCOL Open Access

Planned development and evaluation protocol of two versions of a web-based computer-tailored nutrition education intervention aimed at adults, including cognitive and environmental feedback

Linda Springvloet[1*†], Lilian Lechner[2†] and Anke Oenema[1†]

Abstract

Background: Despite decades of nutrition education, the prevalence of unhealthy dietary patterns is still high and inequalities in intake between high and low socioeconomic groups still exist. Therefore, it is important to innovate and improve existing nutrition education interventions. This paper describes the development, design and evaluation protocol of a web-based computer-tailored nutrition education intervention for adults targeting fruit, vegetable, high-energy snack and fat intake. This intervention innovates existing computer-tailored interventions by not only targeting motivational factors, but also volitional and self-regulation processes and environmental-level factors.

Methods/design: The intervention development was guided by the Intervention Mapping protocol, ensuring a theory-informed and evidence-based intervention. Two versions of the intervention were developed: a basic version targeting knowledge, awareness, attitude, self-efficacy and volitional and self-regulation processes, and a plus version additionally addressing the home environment arrangement and the availability and price of healthy food products in supermarkets. Both versions consist of four modules: one for each dietary behavior, i.e. fruit, vegetables, high-energy snacks and fat. Based on the self-regulation phases, each module is divided into three sessions. In the first session, feedback on dietary behavior is provided to increase awareness, feedback on attitude and self-efficacy is provided and goals and action plans are stated. In the second session goal achievement is evaluated, reasons for failure are explored, coping plans are stated and goals can be adapted. In the third session, participants can again evaluate their behavioral change and tips for maintenance are provided. Both versions will be evaluated in a three-group randomized controlled trial with measurements at baseline, 1-month, 4-months and 9-months post-intervention, using online questionnaires. Both versions will be compared with a generic nutrition information control condition. The primary outcomes are fruit, vegetable, high-energy snack and fat intake.

Discussion: The evaluation study will provide insight into the short- and long-term efficacy of both intervention versions in adults. Additionally, differences in the efficacy among high- and low-educated people will be examined. If these interventions are effective, two well-developed interventions will become available for the implementation and promotion of healthy dietary patterns among both high- and low-educated adults in the Netherlands.

Trial registration: Dutch Trial Registry NTR3396.

Keywords: Environmental feedback, Self-regulation, Computer tailoring, Nutrition education, Fruit intake, Vegetable intake, Fat intake, Snack intake

* Correspondence: linda.springvloet@maastrichtuniversity.nl
†Equal contributors
[1]Department of Health Promotion, School for Public Health and Primary Care (CAPHRI), Maastricht University, P.O. Box 616, Maastricht 6200, MD, the Netherlands
Full list of author information is available at the end of the article

© 2014 Springvloet et al.; licensee BioMed Central Ltd. This is an Open Access article distributed under the terms of the Creative Commons Attribution License (http://creativecommons.org/licenses/by/2.0), which permits unrestricted use, distribution, and reproduction in any medium, provided the original work is properly cited. The Creative Commons Public Domain Dedication waiver (http://creativecommons.org/publicdomain/zero/1.0/) applies to the data made available in this article, unless otherwise stated.

Background

Unhealthy dietary intake patterns are an important risk-factor for multiple chronic diseases, like cancer, cardiovascular diseases (CVD) and diabetes mellitus type II [1]. Morbidity from conditions such as CVD and obesity could be reduced when more people would adopt a healthy diet [2].

The World Health Organization recommends consuming at least 400 grams of fruit and vegetables a day, and to control fat intake to a maximum of 30% of daily energy intake (E%) [3], with a maximum of 10E% from saturated fat [4]. The needed energy intake ranges from 1,600 to 2,400 kcal for women and from 2,000 to 3,000 kcal for men, depending on age and energy expenditure [5]. However, in most developed countries the consumption levels of fruit and vegetables are below [4], and those of fat [4,6] and energy [7] above these recommended levels.

The Dutch recommendations are to consume at least two pieces of fruit and 200 grams of vegetables per day [2,8] and the adequate intake range for total fat is set at 20 to 40E%, with a maximum of 10E% for saturated fatty acids [2]. Although the exact recommendations for energy intake depend on age and energy expenditure, the average guideline for daily energy intake is 2,000 kcal for adult women and 2,500 kcal for adult men [9]. The mean daily consumption of vegetables of Dutch adults is, however, only 130.1 grams and the mean habitual fruit consumption is only 117.4 grams a day. In all age groups, the median habitual total fat intake is above 20E% and 8–10% even have a habitual total fat intake above 40E%. For saturated fat, 88–92% of the Dutch population had a higher habitual intake than recommended. In addition, Dutch males have a too high energy intake [2].

Unhealthy dietary patterns are prevalent in all socioeconomic groups, but the prevalence is highest among people with a low socioeconomic status (SES) (e.g. [2,10-13]). Low SES groups consume less fruit and vegetables [2,10-12] and have a higher intake of energy [12,13] and fat [2] compared to people with a high SES. Inequalities in some diseases, such as CVD and some forms of cancer, may be partially due to SES differences in dietary intake [14].

Despite decades of nationwide nutrition education, the prevalence of unhealthy dietary patterns is still high and inequalities in intake between high and low socioeconomic groups still exist. It is, therefore, important to innovate and improve existing nutrition education interventions.

Because unhealthy dietary patterns are prevalent among a large group of the population, it is important that a nutrition education intervention reaches a large number of people, preferably at relatively low costs. Computer tailoring is a strategy that has the potential to reach a large population with individualized feedback at relatively low costs [15]. In computer-tailored interventions, health information can be adapted to the specific needs and characteristics of a person [16,17]. Several reviews [15,18,19] have shown that (web-based) computer-tailored interventions may have positive effects on the intake of fruit, vegetables and fat, compared to general or no information. Computer-tailored interventions have also been found to be effective among low SES groups [20]. Web-based computer-tailored interventions can potentially have a higher reach than print-delivered versions, especially since Internet penetration rates in the Netherlands are high [21,22], including among low SES groups [22]. Therefore, in this study we chose to develop a web-based computer-tailored nutrition education intervention.

Effect sizes of existing computer-tailored interventions aimed at dietary behaviors are, however, often small [15]. Traditional nutrition education interventions have mainly targeted motivational determinants, such as attitude and self-efficacy. Although motivation is an important first step in the behavior change process, it is not likely that motivation alone will lead to sustained behavior change [23-25]. This 'motivation-only' approach neglects, for example, the important volitional phase of the behavior change process [23,25]. In the motivational phase of behavior change, people form intentions to adopt a healthful diet, but these are often not translated into action. The volitional phase of behavior change focuses on bridging this gap between intention and action and thus facilitates actually making changes. In order to achieve sustained behavior change, it is important that changes in dietary intake are maintained over time. This requires self-regulation skills, which are often not targeted in traditional nutrition education interventions. Self-regulation [26] motivates and enables people to achieve self-set goals. Targeting volitional and self-regulation processes may increase effect sizes of interventions and may help achieve sustained behavior change.

Besides cognitive determinants and self-regulation principles, environmental factors are likely to be important drivers of dietary behavior, as stated in the Social Cognitive Theory [27] and the EnRG framework [28]. Traditional interventions, however, do not take into account the environmental factors that can facilitate or hinder individuals to be able to act on their intentions [25,29]. Therefore, addressing environmental-level factors in a computer-tailored intervention may contribute to improving the effect sizes of computer-tailored interventions.

This paper describes the systematic development and the content of two versions of a web-based computer-tailored nutrition education intervention, in which these

novel elements will be incorporated. By detailing the intervention development and design, we comply with recent calls for a specific intervention description, which increases the transparency of intervention content and improves the options for replication [30].

Methods/design

The development of the intervention was guided by the Intervention Mapping (IM) protocol, which ensures that the intervention is theory-informed and evidence-based and maximizes the likelihood of effectiveness [31]. The IM protocol distinguishes six steps to intervention development, implementation and evaluation. The present paper is specifically focused on the steps to intervention development.

Step 1: needs assessment

Based on the needs assessment, of which a summary is provided in the introduction section, the intake of fruit, vegetables, energy and fat were identified as target behaviors. In addition, people with a low educational level, as an indicator for SES, were identified as an important risk group. To limit participant burden and to be able to develop brief and easy-to-use intervention modules, we decided not to focus on total energy intake, but to focus on the intake of high-energy snacks, since these have been found to be an important contributor to excess energy intake [32]. A change in high-energy snack intake can therefore result in a considerable change in energy intake. Therefore, the following overall intervention goal was set: increasing fruit and vegetable consumption and decreasing high-energy snack and (saturated) fat intake among Dutch adults, in both high- and low-education groups.

Step 2: performance objectives, determinants and change objectives

Dietary intake is a complex behavior that cannot be changed directly, but that results from a number of specific actions that have to be taken, such as 'Deciding to increase fruit consumption'. These more specific actions can be translated into 'performance objectives' (POs) [31], which are the target behaviors (e.g. 'Monitoring fruit consumption') that people have to perform in order to reach a behavioral goal (e.g. 'Increase fruit consumption').

Self-regulation theory was used as one of the central models for this intervention and guided the formulation of the POs. According to Maes and Karoly [33,34] self-regulation occurs in multiple phases: a) a pre-action phase, in which the existence of a problem, such as low fruit consumption, is recognized, an intention to change is formed and a goal and action plan are set; b) an action phase, in which people execute their plans and try to reach their intended behavioral goal and; c) an evaluation phase, in which people evaluate their behavior change and, depending on the evaluation results, maintain or adapt their goals and action plans.

For each of the four dietary behaviors targeted in the intervention (i.e. fruit, vegetables, high-energy snacks and fat) POs were formulated, based on the three phases of self-regulation. The POs for the target behavior fruit consumption are illustrated in Table 1. The POs for vegetables, high-energy snacks and fat are similar and are therefore not presented in this paper.

The next step of this second phase of the IM protocol was to analyze determinants of the selected target behaviors [31,35]. To identify the important and changeable individual- and environmental-level determinants we conducted a review of the empirical literature on determinants of dietary behavior in general and the specific target behaviors, i.e. intake of fruit, vegetables, high-energy snacks and fat. In addition, we identified potentially relevant individual-level determinants from motivational- and volitional theories, such as self-regulation theory [26], the Precaution Adoption Process Model [36] and the Theory of Planned Behavior [37].

Based on the Precaution Adoption Process Model [36,38], awareness of one's own dietary intake was identified as a determinant of dietary behavior in general. The Theory of Planned Behavior (TPB) variables – intention, attitude, self-efficacy, perceived behavioral control and subjective norms – were also found to be associated with dietary behavior in general [39]. General environmental-level factors that were identified in the literature were perceived availability and perceived price differences between healthy and unhealthy food options [40].

For both fruit and vegetable consumption the following individual-level factors were identified: knowledge [10,41,42], self-efficacy [41,43,44], attitudes [41], intention [41,45], self-regulation [43], action and coping planning [46] and habit [42,45,47,48]. Based on two Dutch focus group studies taste was also identified to be important [47,48]. Environmental-level factors that were identified for both fruit and vegetable consumption were: perceived availability [11] and perceived costs of fruit and vegetables [11,49]. For fruit, the availability of fruit in the home environment was also found to be related to intake [49,50].

Determinants of the intake of energy or high-energy snacks were under studied and therefore only a few individual-level factors have been found: self-efficacy, attitude, intention [51] and taste [52].

Fat intake was shown to be associated with self-efficacy [43], attitude [53], self-regulation [43] and habit [53]. No specific environmental-level determinants were identified for fat intake.

In addition, some differences in determinants between high and low SES groups were found. Relatively high

Table 1 Performance objectives for fruit consumption

Self-regulation phase	Performance objectives
**Phase I: pre-action**	PO1: Individuals monitor their own fruit consumption.
	PO2: Individuals compare their fruit consumption with the recommendations.
	PO3: Individuals recognize the importance of increasing their fruit consumption.
	PO4: Individuals decide to increase their fruit consumption.
	PO5: Individuals prepare their behavior change:
	-PO5a: Individuals set a challenging, but feasible goal to increase fruit consumption
	-PO5b: Individuals make action plans for their behavior change.
**Phase II: action**	PO6: Individuals start changing their behavior: they eat more fruit and/or eat fruit more often.
	PO7: Individuals keep track of situations that caused failure.
**Phase III: evaluation**	PO8: Individuals evaluate the achievement of their goals:
	When behavioral goal has not been reached, individuals choose to:
	-PO8a: Continue with their behavioral goal (depending on reasons of failure);
	-PO8b: Adjust their action plan for the same behavior change goal;
	-PO8c: Adjust their goal for fruit consumption;
	-PO8d: Set a new behavioral goal for another target behavior (i.e. vegetables, high-energy snacks or fat)
	When behavioral goal has been reached, individuals choose to:
	-PO8e: Adjust their behavioral goal, in order to improve their fruit consumption even more (for example, when their goal was to eat one piece of fruit instead of zero, they can change their goal to eating two pieces of fruit);
	-PO8f: Set a behavioral goal for another target behavior (i.e. for vegetables, high-energy snacks or fat).
	PO9: Individuals maintain the increase in fruit consumption:
	-PO9a: Individuals keep monitoring their fruit consumption;
	-PO9b: Individuals take action when they diverge from behavioral goal.

prices of healthy food products are suggested to be more important for low SES groups [12,47,54]. In addition, the relative importance of price in food choices is associated with a lower consumption of fruit and vegetables and a higher intake of energy-dense food and may therefore mediate the effect of SES on the intake of fruit, vegetables and energy-dense foods [12]. Another study showed that perceived affordability, perceived food availability and accessibility (almost fully) mediate the association between SES and diet [11]. In addition, adolescents in high SES families perceive a higher accessibility of fruit and vegetables at home [50].

After the determinant analysis we defined change objectives (COs), which are the most direct target behaviors of the intervention. COs were defined by crossing the POs with the selected determinants [31]. For example: crossing the PO 'Individuals decide to decrease their high-energy snack intake by substituting it for a lower-energy snack' with the determinant 'perceived availability' gives the CO 'Individuals perceive lower-energy snacks as highly available in their supermarket'. Examples of change objectives for the target behavior fruit consumption are given in Table 2.

Step 3: theory-based methods and practical applications

In the third step of the IM protocol we selected theoretical methods and practical applications to modify the determinants of the dietary target behaviors, in order to achieve the change and performance objectives. A method is a theory-based technique that influences a determinant and can be delivered via a practical application. When translating theoretical methods into practical applications, it is important to meet the conditions under which the theory and method are effective (i.e. 'parameters for use') [31,35]. Modeling, for example, is a theoretical method derived from the Social Cognitive

Table 2 Selection of change objectives for fruit consumption

Performance objective	Determinants			
	Awareness	Attitude	Self-efficacy	Perceived availability
PO1: Individuals monitor their fruit consumption	Individuals are aware of their own fruit consumption			
	If applicable: individuals recognize the problem of their own low fruit consumption			
PO4: Individuals decide to increase their fruit consumption		Individuals express positive attitude beliefs towards increasing their fruit consumption	Individuals identify barriers to increasing their fruit consumption	Individuals know which fruit products they can buy in their supermarket
		Individuals can explain the health benefits of increasing their fruit consumption	Individuals are confident that they can cope with barriers	Individuals perceive fruit as highly available in their supermarket

Theory [27] that can be used to modify self-efficacy. A practical application for delivering modeling to the participants is including role model stories quoting how peers coped with a certain barrier. Modeling is only effective when the target group can identify with the model, when a coping model is presented and when a reinforcement of the behavior is visible [31,35]. The determinants, theoretical methods and practical applications per self-regulation phase are described in more detail below. The design is the same for each target behavior (i.e. fruit, vegetables, high-energy snacks and fat), unless stated otherwise, and therefore, on general, no distinction between the target behaviors is made in the description. An overview of the methods and applications applied in the intervention, divided into the three self-regulation phases, is shown in Additional file 1 (Table S1).

Phase I: Pre-action

In the pre-action phase of self-regulation, the motivational determinants are the most important to target [26].

Knowledge

Knowledge is targeted by providing general information in the form of short facts about the target behavior and information on the consequences of not complying with the guideline regarding the target behavior [30].

Awareness

Tailored behavioral, normative and comparative feedback [55] are used to increase awareness of one's intake of fruit, vegetables, high-energy snacks and/or fat.

Participants can complete a detailed questionnaire assessing their consumption levels (i.e. monitoring behavior), based on which textual tailored feedback is provided on the consumption level (behavioral feedback), normative feedback on how one's own consumption compares to the guidelines regarding the target behavior and, when a person has a lower intake than peers of the same age and gender, comparative feedback on how consumption of the target behavior compares to the consumption of peers (i.e. providing information about personal risk [31]).

Attitude

Methods used to influence participants' attitudes are belief selection, persuasive communication and modeling [31,56].

Participants can choose two advantages and disadvantages that are most important to them from a predefined list of behavior-specific cognitive and affective advantages and disadvantages (i.e. belief selection). Examples of attitude items are: 'Eating more fruit is good for my health' and 'When I eat lower-energy snacks I feel less energetic'. Subsequently, tailored feedback is provided in order to reinforce the selected advantages and to weaken the selected disadvantages, or to correct incorrect assumptions (i.e. persuasive communication). In the feedback on disadvantages of the target behavior, modeling is incorporated via peer stories. A peer describes, for example, that he or she thought he or she would feel less energetic when eating lower-energy snacks but that the opposite turned out to be the case when trying to eat lower-energy snacks. For fruit and vegetable consumption a 'taste-test' is incorporated in the feedback on, for example, the disadvantage 'I don't like the taste of fruit'. Participants who select this disadvantage can complete a test to identify their preferred taste (i.e. sweet, sour, bitter or combined) after which information is provided on fruit that matches their preferred taste.

Environmental information is incorporated in the tailored feedback on disadvantages by providing tailored information on the availability and/or price of healthy food products in the supermarket where the participant usually does the shopping. When a person, for example, selects the disadvantage 'Vegetables are expensive', information about the

price of vegetables in the usually visited supermarket is provided.

The fat module does not include methods that target attitude.

Self-efficacy

The methods used to target self-efficacy in the first self-regulation phase are prompting identification of barriers [30], persuasive communication [31,56], providing instructions [30], modeling [56], goal setting [30,57] and action planning [31,58].

In the first part of the self-efficacy section participants select barriers from a predefined list (i.e. barrier identification), such as ‘I can’t resist the temptation to eat a high-energy snack’ and ‘I don’t know how I can eat more fruit’. After choosing the barriers, tailored feedback with solutions to overcome them is provided (i.e. persuasive communication and providing instructions). In this feedback, modeling is incorporated via peer stories. A peer gives, for example, tips on how to eat more fruit, even in the face of the identified barrier.

Environmental information is incorporated in the tailored feedback on barriers by providing tailored objective information on the availability and/or price of healthy food products in the usually visited supermarket. A predefined barrier for fruit consumption is, for example: ‘Fruit is not widely available’. In the feedback on this barrier, tailored objective information about the availability of fruit is provided.

In the second part of the self-efficacy section participants select two predefined situations in which they think it can be more difficult to perform healthy behavior, such as ‘When there is no delicious fruit available’ and ‘When I eat in a restaurant’. For these difficult situations tailored feedback with practical solutions is provided (i.e. persuasive communication and providing instructions). Modeling is incorporated in this feedback by providing stories on how peers found solutions to cope with the difficult situation.

The last part of the self-efficacy section is aimed at goal setting and action planning, in order to translate the intention, which is formed in the previous parts, into action. Goal setting has the potential to facilitate (dietary) behavior change [59,60] and leads to higher performance levels when goals are specific and challenging or difficult [56,61]. To give participants sufficient freedom of choice, and to make it fit with self-regulation [62], goal setting is incorporated in the intervention as an open-ended format, meaning that participants are free to formulate their own goal in provided text boxes. In order to guide participants, short instructions and examples of goals are provided.

Once the goal is set, participants can formulate an action plan on how to reach their goal, in the form of implementation intentions, which are if-then plans that specify both the behavior that one will perform in the service of goal achievement and the situational context in which one will act [58], for example: ‘If I am having lunch, then I am going to eat an apple’. Implementation intentions are incorporated in the intervention as an open-ended format and participants can specify, in provided text boxes, when, where and how they are going to act to reach their intended goal. For example: for high-energy snack intake participants first specify when they want to perform their new behavior (e.g. ‘When I am watching television’) and subsequently what they are going to do instead of eating the high-energy snack (e.g. ‘Then I am going to take a short walk’), or how they are going to substitute the high-energy snack (e.g. ‘Then I will eat fruit’).

The fat module does not include methods that target self-efficacy.

Availability and accessibility of healthy food products in the home environment

The methods used to target the home environment are monitoring, persuasive communication [31,56], providing instructions [30] and (as a result) creating a more supportive environment.

Participants are asked whether they always have fruit, vegetables or high-energy snacks available at home and where they store these products (i.e. monitoring). Tailored feedback is provided on whether participants already arrange their home as a supportive environment or whether they can make some improvements (i.e. persuasive communication). Practical suggestions are provided in order to make the healthy food products more available and accessible and the unhealthy food products less available and accessible, for example: ‘Do not always have high-energy snacks available at home’, and ‘If you do have high-energy snacks available at home, store these in a place where you don’t always see them and put them behind the more healthy products’. As a result, participants are stimulated to rearrange their home environment if necessary.

The fat module does not include methods that target the home environment.

Perception of availability and price of healthy food products in the supermarket

The method used to target the perception of availability and price of healthy products (i.e. fruit, vegetables, low-energy and low-fat products) in the supermarket is tailored objective information, as already briefly described in the description of attitude and self-efficacy. This feedback is provided in response to specific attitude and self-efficacy beliefs, but also in a separate section, where participants again can read information on the

availability and price of healthy food products in the supermarket they usually visit.

Phase II: action

In the action phase participants try to achieve their behavioral goal, by performing the specific actions that they have planned. The important determinants in this phase are awareness of the progression toward a successful behavior change and self-efficacy. Participants are asked to monitor every day for themselves whether they achieved their goal (i.e. monitoring [30]) and which difficult situations they encountered (i.e. prompting identification of barriers [30]). This information can be used by the participants as feedback on the achievement of the goals and plan, and is used as input for the evaluation phase.

Phase III: evaluation

Awareness of the progression of the behavior change

The first method to target awareness in the evaluation phase is monitoring the progression of the target behavior. Participants can state for each day of the past week whether or not they achieved their behavioral goal (i.e. monitoring progression of behavior change/prompt review of behavioral goals [30]). Based on this monitoring, tailored feedback on performance [30,63,64] is provided. For fruit and vegetable consumption, participants also report their fruit or vegetable consumption in the past week. Fruit and vegetable consumption is then compared with the consumption during the previous visit to the intervention and participants receive feedback on their progress in fruit and vegetable consumption. The snack and fat module did not include monitoring of intake, because the assessment questionnaires were quite long.

Attitude

A decisional balance is used to target attitude in the evaluation phase. Participants who indicate they have not achieved their behavioral goal because they were not motivated are stimulated to make a decisional balance by specifying, in provided text boxes, the advantages and disadvantages of changing the target behavior. Subsequently, an overview of this balance is provided and participants are stimulated to review the balance. After seeing their decisional balance, participants can indicate whether they are motivated to make a second attempt or whether they want to choose another target behavior.

Self-efficacy

To target self-efficacy in the evaluation phase, coping planning is used [65]. Participants who indicate they have not achieved their behavioral goal because of circumstances can select which circumstances hindered them to perform their intended actions from a predefined list (e.g. ‘I had a very strong desire to eat something else’) or describe a situation via an open-ended question. Subsequently participants are stimulated to make a plan to overcome the same situation the next time (i.e. coping planning). Coping planning is provided in an open-ended format, in which participants can state their own solution to overcome the difficult situation.

In addition, all participants can state which difficult situations they expect in the following weeks. Participants can choose a situation from a predefined list (e.g. ‘When the fruit I want to eat is not available’) or specify an expected situation via an open-ended question, and are subsequently stimulated to make a plan to overcome this situation (i.e. coping planning, provided in an open-ended format).

Next, participants are asked whether they are confident of achieving their goal in the next time period. When they are not confident, they have the opportunity to lower their goal in order to make it more feasible. When they are confident they have the opportunity to increase their goal in order to make it more challenging.

The last method to target self-efficacy in the evaluation phase is providing instructions [30] and information about how participants can maintain their new behavior. Participants are instructed to follow a specific sequence of actions, for example: ‘Monitor your fruit consumption’, ‘Make a plan for a new difficult situation’, and ‘Adapt plans that are not effective enough’. Also, instructions on what to do when one relapses to old habits and some practical suggestions on how to maintain the new behavior are provided, such as ‘Make sure you always have fruit available at home’.

Step 4: development of the online tailored intervention

The web-based, computer-tailored intervention we developed is called ‘Bewust eten, hoe doe je dat?’ (‘Conscious eating, how do you do it?’) and consists of four modules: fruit, vegetables, high-energy snacks and (saturated) fat. The methods, as described in the previous part, are incorporated into two versions of the intervention. One version, the basic version, is only targeted at the individual cognitions and volitional and self-regulation processes, and therefore only includes the methods on knowledge, awareness, attitude and self-efficacy. The other version, the plus version, additionally targets the environmental factors and therefore also includes methods on the availability and accessibility of healthy food products in the home environment and the perception of availability and price of healthy food products in the supermarket. The technical development, involvement of the target group and the outline and sequence of the final intervention are described in more detail below.

Development of the online tailored intervention

The web-based, computer-tailored intervention is developed using the TailorBuilder software (OSE, the Netherlands), which is suitable for generating tailored feedback based on assessment questionnaires, tailoring algorithms and feedback messages. The intervention is delivered via a website (see Figure 1) where participants can log in with their personal codes.

The individual feedback on the intake of fruit, vegetables, high-energy snacks and fat is based on existing computer-tailored nutrition education interventions [66,67]. The (feedback on) beliefs and barriers included in the intervention are partly based on these interventions, but also new beliefs and barriers are included, based on the input of a consumer panel (see Involving the target group during intervention development).

The environmental information, which consists of the availability and price of selected fruit, vegetables, lower-energy snacks and lower-fat products, is collected via observations in the local supermarkets (n = 58). A research assistant audited the cooperating supermarkets (n = 31) and observed the availability and price of selected products. Subsequently, the observed local supermarket information was incorporated into the computer-tailored intervention. Unfortunately, not all supermarkets agreed with the observations. For the non-cooperating supermarkets (n = 27, of which 23 are affiliates of three large supermarket chains who did not wish to participate), general information on the availability and price of healthy food products in supermarkets is provided to the participants. To keep product prices up to date, supermarket representatives were asked to send price updates every month. Most of the time, however, the research assistant had to re-audit the supermarket

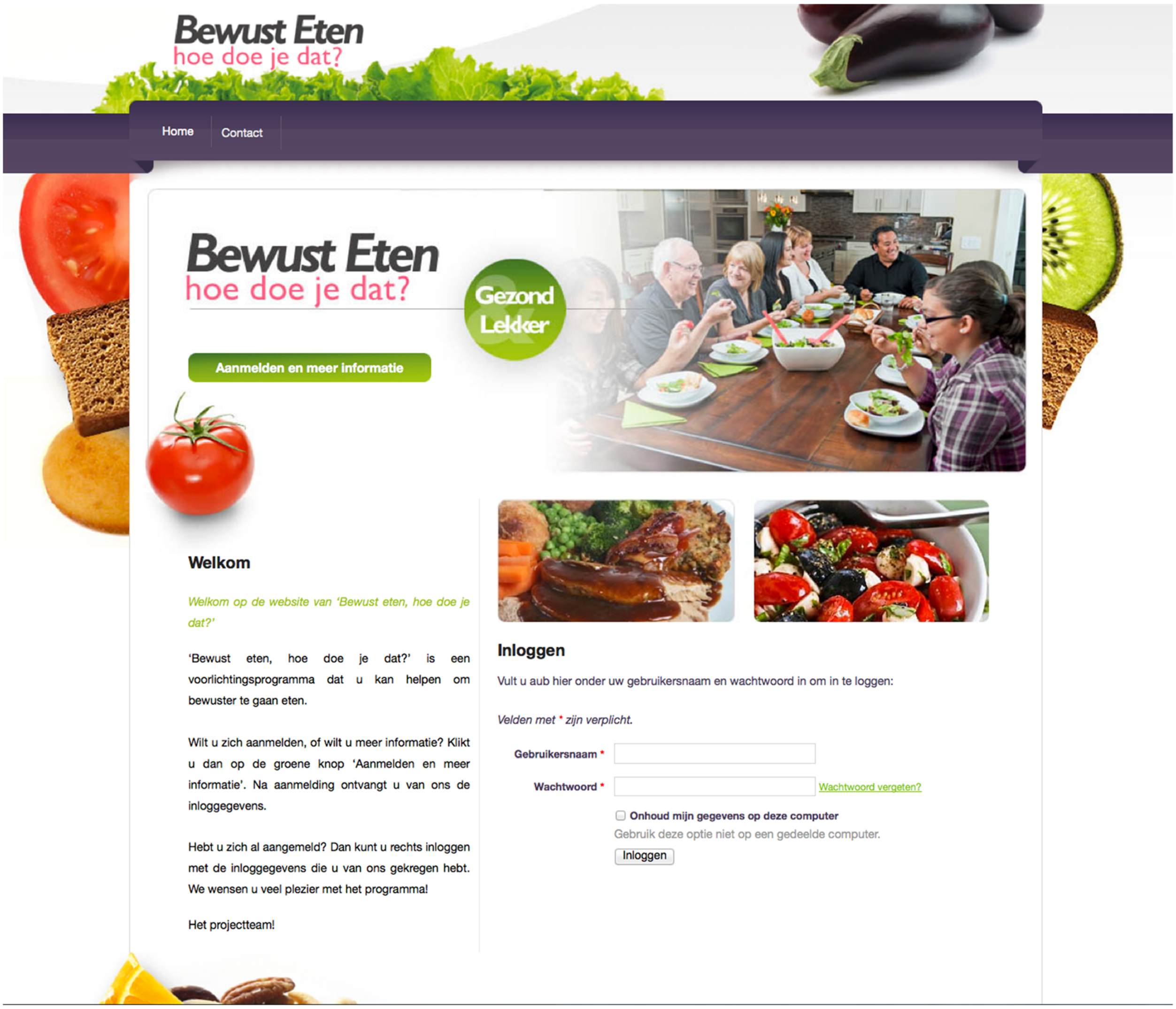

Figure 1 Print screen of the homepage of the intervention.

to update prices. In addition, information on websites of supermarkets is used to collect information about the availability and price of products and to update the information.

Involving the target group during intervention development

Representatives of the target group, consisting of high- and low-educated adults aged 20–65 years, were involved during the developmental phase in two ways: via a consumer panel and a pretest.

Consumer panel

The consumer panel consisted of 55 people from the target group (mean age 51.31 years (SD = 12.43); 63.6% women; 40% low-educated) who were recruited via spreading flyers in low SES neighborhoods and recruiting people in shopping malls.

Members of the consumer panel were contacted multiple times via e-mail or phone. They were asked to provide input on, for example: advantages and disadvantages of the target behaviors; barriers to performing the target behavior; and locations in the home environment to store food products. They were also asked to provide feedback on ideas about the intervention name, appreciation of the recruitment folder and on how we could reach participants for the evaluation study. With the help of the consumer panel we were able to make the intervention fit the target group and to address beliefs and barriers that are salient in the target group.

Pretest

A first version of both versions of the intervention was pretested in order to identify points for improvement. A total of 44 persons participated in the pretest of the basic version. A qualitative and a quantitative pretest were conducted.

Twelve people (mean age 41.9 years; 75% women; 50% low educated) participated in the qualitative pretest. During this pretest participants worked through the intervention in the presence of a researcher, who stimulated participants to think aloud and asked in-depth questions to check the comprehensibility of the information, such as ‘Can you explain in your own words what you have read?’. Afterwards, participants filled out a short questionnaire to express their appreciation of the intervention and were stimulated to explain their answers. The qualitative pretest was also used to pretest the homepage of the website (see Figure 1); participants were asked to give feedback in terms of attractiveness, clarity and comprehensibility.

Twenty-eight participants (mean age 48 years; 64.29% women; 35.7% low-educated) participated in the quantitative pretest of the basic version of the intervention. Participants worked through the intervention at home and filled out a questionnaire that measured, amongst others, appreciation (e.g. ‘What score do you give this intervention?’), user-friendliness (e.g. ‘This intervention was easy to use’) and comprehensibility (e.g. ‘The information was easy to understand) of the intervention and specific components.

The basic version of the intervention was appreciated quite well (average rating = 7.98 (SD = 1.06)) and the information was rated as useful, interesting, easy to understand, personally relevant and user-friendly. The time it took to work through the intervention was rated as being acceptable. Points for improvement were the attractiveness of the layout and the length of the texts. The texts were too long and participants tended not to read all the texts in their entirety. Therefore, the texts were made shorter and more concise. The less positive rating of the attractiveness of the layout was mostly because the intervention was not pretested in its final layout. After finalizing the layout, different members of the consumer panel rated the new design as being attractive. All participants who were shown the website appreciated the layout of the website and thought it was clear how to log in to the website.

A group of six participants (mean age 46.2 years; 83.33% women; 16.67% low educated) pretested the plus version of the intervention and 15 members of the consumer panel provided feedback on an example of price and availability information for lower-energy snacks. The plus version was scored with a 7.7 (SD = 0.98). The most important results were that the environmental information was seen as being too independent from the other parts of the intervention and therefore it was not clear how it related to the tailored feedback on other determinants. Also, two participants thought the information was too commercial and thought the goal was to show supermarket advertisements. The first comment was solved by incorporating environmental information in the attitude and self-efficacy sections in order to relate it to the other information. To solve the commercial look of the environmental information, we accompanied the information about availability and prices with more textual information in order to explain why this information is relevant.

Scope and sequence of the intervention

Each module (i.e. fruit, vegetables, high-energy snacks and fat) consists of three sessions of approximately 30 minutes that can be worked through with a two-week interval between each session (taking six weeks in total). All sessions consist of multiple sections in which specific determinants are targeted. A general outline of the intervention is shown in Figure 2. The content and sequence of the sessions are described below. The content and sequence for the modules about vegetables, fruit and high-

energy snacks are the same. The module about fat intake, however, does not include the attitude and self-efficacy sections in the first session, in order to limit participant burden, since the assessment of fat intake is quite long.

First session

After receiving their log-in code and password participants can log in to the intervention via a special website (see Figure 1). After reading a short introduction to the intervention participants can choose which target behavior they want to address first.

The first session is aimed at the pre-action phase of self-regulation and starts with increasing knowledge of the target behavior: participants can self-tailor the information by choosing the topics of their interest. The next section is aimed at awareness: participants fill out an assessment questionnaire on the target behavior and receive tailored feedback. When participants already comply with the dietary guidelines, they are provided with the opportunity to choose another target behavior. When they do not comply, they enter the attitude section.

The attitude section is not compulsory; participants who indicate that they think changing the target behavior is important are given the opportunity to skip this section. The attitude section first addresses the advantages and subsequently the disadvantages regarding (changing) the target behavior. Subsequently, self-efficacy is targeted, which is also not compulsory and can be skipped (partly or completely) when participants are already confident that they can change the target behavior. The first part of the self-efficacy section is aimed at barriers and the second part at difficult situations.

The plus version has two extra sections after the self-efficacy section. First, the home environment regarding the target behavior is targeted and subsequently objective information is provided about the availability and price of healthy food products in the supermarket where the participant tends to do his or her shopping.

The first session ends with setting a goal and formulating an implementation intention. After formulating the behavioral goal and implementation intention, an overview of this goal and implementation intention is provided on screen. The goal and implementation intention can be printed and are also saved on the website and can be reviewed when revisiting the website. At the closing page of the first session, participants can choose another target behavior or close the intervention.

After the first session, participants can execute their plan(s) for two weeks in order to achieve their goal(s) (i.e. the action phase). After two weeks, they will be invited by e-mail to return to the intervention for the second session.

Second session

The second session is aimed at the (short-term) evaluation of the behavior change and starts with targeting

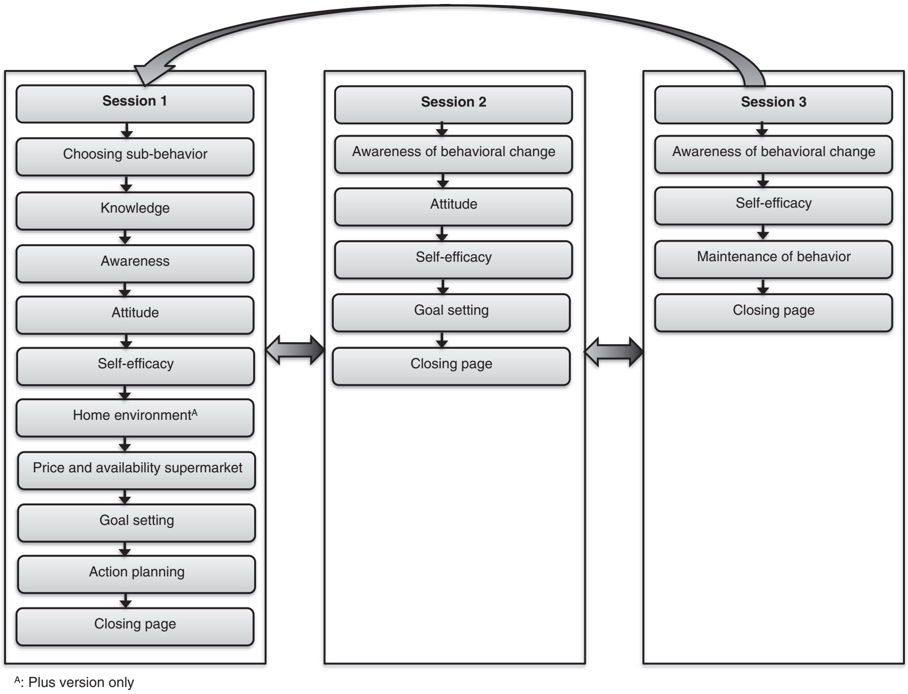

Figure 2 Overview of the sequence of the intervention.

the awareness of the behavior change: participants can monitor their goal achievement and receive tailored feedback.

Participants who did achieve their goal on all days are complimented and forwarded to the 'expected difficult situations' part. Participants who did not achieve their goal on all days enter either the self-efficacy section to identify encountered difficult situations and to formulate coping plans, or the attitude section to see whether they are still motivated. Participants who indicate that they are not motivated to make a second attempt are forwarded to a page where they can choose another target behavior.

All participants, except participants who are redirected to another target behavior, subsequently enter the expected difficult situations part in which a coping plan is formulated. The last part of the self-efficacy section is aimed at goal setting and provides participants the opportunity to adapt their goal.

The second session ends with an overview of the coping plan(s) and (adapted) goal(s), which can be printed and is also saved on the website. At the closing page of the second session, participants can choose (to evaluate) another target behavior or close the intervention. After the second session, participants again execute their plan (s) for two weeks and will then be invited by e-mail to return to the intervention for the last session.

Third session

The last session is aimed at long-term maintenance. The first part of the third session is more or less the same as the second session: monitoring the performance in the past week and, if applicable, getting insight into which situations caused failure, and subsequently making coping plans for encountered and expected difficult situations. In addition, the last session provides information and instructions on how participants can maintain their new behavior.

At the closing page of the second session, participants can choose (to evaluate) another target behavior or close the intervention.

Evaluation plan

The first goal of the evaluation is to examine the efficacy of both versions of the intervention compared to generic nutrition information. It is hypothesized that after exposure to the intervention participants in both intervention conditions will have a higher increase in fruit and vegetable consumption and a higher decrease of high-energy snacks and fat (i.e. the primary outcome measures) compared to the control condition. In addition, it is expected that both versions of the intervention will result in more favorable values for the secondary outcome measures (i.e. self-reported body mass index (BMI), general self-control and general self-regulation) and mediating variables (i.e. motivational determinants, habit, action and coping planning, perceived availability and price of healthy food products in the supermarket and availability of healthy food products at home) compared to the control condition. We will also examine whether there are differences in the efficacy of both versions of the intervention between high- and low-educated adults. We expect that the plus version is more effective for low-educated participants than for high-educated participants, since environmental factors are suggested to be more important among low SES groups (e.g. price [12,47,54]), and to mediate the association between SES and diet [11].

The second goal of the evaluation study is to examine the appreciation and use of the intervention and its components. In addition, the quality of the goals and action plans will be examined.

Design and procedure

A three-group randomized controlled trial will be conducted to study the effects of both versions of the intervention compared to a control group that will receive generic information about fruit, vegetables, high-energy snacks and fat. The program of the control group also consists of three sessions and will be delivered via the same website as the tailored intervention, ensuring the same layout. Measurements will take place at baseline (T0) and one (T1), four (T2) and nine months post-intervention (T3). Before participation, an informed consent has to be signed and returned by post or e-mail. One month after completing the baseline measurement participants will be randomly assigned to one of the three study groups and participants will have access to their assigned program for two months. Two weeks after each intervention visit e-mail reminders will be sent to prompt returning to the intervention. Invitations to fill out the questionnaires will be sent via e-mail. Two and four weeks after the initial invitation e-mail reminders to fill out the questionnaire will be sent.

When participants fill out all questionnaires, they can win an iPad (of which 20 are allotted) or a gift voucher of 20 euros (of which 500 are allotted). The Medical Ethics Committee of the Erasmus Medical Centre in Rotterdam approved the study protocol (NL35430.078.11/ MEC-2010-408). The trial is registered in the Dutch Trial Registry (NTR3396).

Recruitment of participants

A total of 2,000 adults will be recruited for participation in the study; 900 with a high educational level and 1,100 with a lower educational level. The required number of participants is based on a power calculation, in which 1,400 participants would be sufficient to detect a small

intervention effect, with a power of 0.80 and a significance level of $p < 0.05$. To account for a drop out of 10% of the participants between each measurement, 1,800 participants need to be recruited into the study. To take into account a potentially higher drop out in the lower educational group, we will recruit 200 extra participants in this specific group.

Participants will be recruited in five cities in the south of the Netherlands: Heerlen, Roermond, Venlo, Venray and Weert. The choice to limit the recruitment to these cities was made in order to be able to collect the environmental information needed for the plus version of the intervention. These five cities complied with the inclusion criteria that were defined for cities: having both high and low SES neighborhoods, having at least 25,000 inhabitants, not having too many (small) supermarkets and the municipality should be willing to provide a random selection of home addresses within the cities for recruitment purposes. The main recruitment method will be sending personal letters to randomly selected home addresses, but Facebook advertisements, advertisements in (local) newspapers, local TV and recruitment in shopping malls will also be used as recruitment strategies. Inclusion criteria for the participants are: being between 20 and 65 years of age, having a sufficient understanding of the Dutch language (in reading and writing) and having access to a computer that is connected to the Internet. Exclusion criteria are: being on a diet prescribed by a physician or dietician, having a medical condition that implies restrictions in eating behavior (e.g. CVD or bowel disease) and not willing to sign an informed consent.

Measurements

Online questionnaires will be used to collect self-reported data on the primary and secondary outcome measures. In addition, the potential mediating variables will be assessed, to be able to investigate the mediating pathways of the intervention effects. A process evaluation will also be conducted to assess appreciation and use of the intervention and the various components. Process measures will be incorporated in the first follow-up questionnaire and will contain questions on perceived attractiveness, user-friendliness, comprehensiveness, usability and perceived personal relevance. Also the appreciation of the environmental feedback and the goal setting and action planning tools will be measured. The use of specific intervention components and the number of visits to the intervention will be measured objectively via web-server registrations. Figure 3 provides an overview of the variables measured at each time point.

Statistical analyses

Repeated measures analyses will be conducted to study differences between the three study groups in changes in the food intake measures (i.e. main effects). Additional analyses will be conducted on secondary and mediating outcome measures. Since five cities were included in this study we will adjust for place of residence by including this variable as a covariate in the analyses. Because people with unfavorable diets probably profit more from the intervention, the efficacy of the intervention will also be assessed among subgroups that do not meet recommended intake levels for each target behavior at baseline. Interaction with educational level will be explored to study whether effects of the intervention differ according to educational level (i.e. interaction effects). To gain insight into the pathways of change, mediation of the intervention effects through the potential mediating variables will be examined with the joint significance test [68]. Descriptive analyses and (multiple) linear and logistic regressions will be conducted to study the appreciation of the intervention, the use of different components and the quality of goals and action plans.

Discussion

This paper describes the development, design and evaluation protocol of two versions of a web-based computer-tailored intervention aimed at increasing fruit and vegetable consumption and decreasing high-energy snack and fat intake. Both versions of the intervention were developed systematically by using the Intervention Mapping protocol [31].

The intervention is innovative compared to existing interventions because it addresses the various phases of self-regulation and targets determinants that play a role in the motivational phase of the behavior change process as well as determinants that are important in the volitional phase of the behavior change process. In addition, in one version tailored feedback is provided on the arrangement of the home environment and the availability and prices of healthy products in the local food environment (i.e. objective information on availability and prices of healthy food products in supermarkets).

Incorporating environmental feedback in a web-based, computer-tailored intervention is a novelty for nutrition education, but it has previously been applied in tailored interventions aimed at promoting physical activity (e.g. [69,70]). Providing tailored environmental feedback for dietary behavior is, however, probably more complicated than for physical activity. In this intervention we provide environmental feedback based on information collected from observations in supermarkets. This approach appeared to be feasible, but very time-consuming. Collecting environmental nutrition information for incorporation in interventions would become more feasible if it was possible to make use of supermarkets' digital databases. Most supermarkets do have such digital databases, but many representatives of supermarkets in our study regions were not

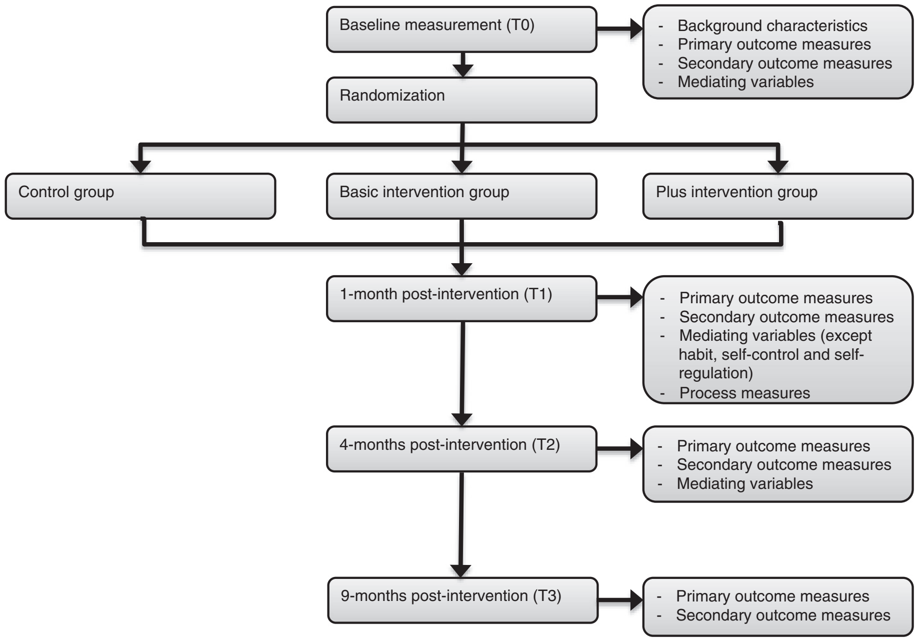

Figure 3 Overview of the procedure of the evaluation study and measurements.

allowed or able to provide us with this digital information. Because prices of food products, especially of fruit and vegetables, are subject to price fluctuations, prices had to be updated several times during the intervention period. Therefore, supermarket representatives were asked to provide us with price updates every two weeks, which proved to be complicated and often new observations were required to update the prices. In some cases information provided on the websites of supermarkets could be used to update the information.

The home environment has been included as a (small) part of the whole intervention. Adapting the home environment may, however, be an intervention in itself. It is a complex behavior that includes multiple behavioral determinants, such as awareness, attitude and self-efficacy, which are not all explicitly targeted in this intervention. The effect evaluation will give insight into whether briefly covering the home environment already contributes to behavior change, or whether a more elaborate approach is needed.

Determinants of high-energy snack intake were under studied and therefore we had to make some assumptions for determinants based on theory and other behaviors. The effect evaluation will give insight into whether targeting the selected determinants is effective in changing high-energy snack intake, or whether other determinants are needed to be included in interventions aimed at reducing high-energy snack intake.

One step of the Intervention Mapping protocol has not explicitly been described in this paper; the adoption and implementation plan [31]. During the intervention development we did anticipate the adoption and implementation of the intervention by involving a municipal health services organization, members of the target group and representatives of supermarkets. Implementing the basic version of the intervention on a large scale will not be very complex, because providing tailored information based on individual and psychosocial determinants can be provided to anyone and is not region-specific. Implementing the version that includes environmental feedback will, however, probably be more difficult. If this version of the intervention is shown to be effective in changing dietary behavior and is thus suitable for implementation on a large scale, more efficient ways to collect the environmental information will be needed. To achieve this, close cooperation with supermarkets is necessary. This may require involving the headquarters of supermarkets, since the approach that we took by contacting the local supermarket managers did not result in active collaboration. If the intervention can be promoted as being an effective intervention, this could be an extra reason for supermarkets to collaborate. Furthermore, involvement of representatives of the municipalities may improve the chance of cooperation from the supermarkets.

Because unhealthy dietary patterns are even more prevalent among people with a lower educational level, we explicitly took this target group into account. To make the intervention suitable for lower-educated participants, this specific group was involved via both the consumer panel and pretest. This way, we were able to take the wishes and needs of this particular group into account, such as keeping the provided information as clear as possible. In addition, also involving high-educated

people during the development of the intervention increases the likelihood that the intervention is attractive and appreciated by all subgroups within the target group. The pretest has already shown that both high- and lower-educated adults appreciated both versions of the intervention, and adaptations for improvement were made.

Although a well-planned, theory- and evidence-based intervention has a higher likelihood of being effective, the efficacy of the intervention still has to be examined. This will be done in a three-group randomized controlled trial, which will give insight into the efficacy of both versions of the intervention compared to a generic nutrition information control group. In addition, differences in the efficacy among high- and low-educated people will be examined. Other studies, for example of van Genugten, van Empelen and Oenema [71], have shown that planning tools are not always used optimally and that formulated goals and plans are not always of good quality. Therefore, we will also examine the quality of the goals and action plans and which intervention elements are used by the participants.

If the evaluation study shows that the two versions of the intervention are effective, then two well-developed interventions will become available for the large-scale implementation and promotion of healthy dietary patterns among both high- and low-educated adults in the Netherlands.

Additional file

Additional file 1: Table S1. Theoretical methods and applications used in the intervention.

Competing interests
The authors declare that they have no competing interests.

Authors' contributions
AO and LL designed and wrote the original study proposal. LS developed the intervention and coordinate the study. AO and LL supervised the development and study. LS drafted the manuscript. AO and LL were involved in revising it critically. All authors read and approved the final manuscript.

Acknowledgements
We would like to thank Karin Hummel for her important role in different parts of the intervention development. The study was funded by ZonMw, the Netherlands Organisation for Health Research and Development (grant number: 200110021).

Author details
[1]Department of Health Promotion, School for Public Health and Primary Care (CAPHRI), Maastricht University, P.O. Box 616, Maastricht 6200, MD, the Netherlands. [2]Faculty of Psychology and Educational Sciences, Open University of the Netherlands, PO Box 2960, Heerlen 6401, DL, the Netherlands.

Received: 18 December 2013 Accepted: 13 January 2014
Published: 17 January 2014

References

1. van Kranen HJ, van Raaij JMA, van Bakel AM: **Wat is de relatie tussen voeding en gezondheid? (What is the relation between nutrition and health?).** In *Volksgezondheid Toekomst Verkenning, Nationaal Kompas Volksgezondheid.* Bilthoven: RIVM; 2013.
2. van Rossum CTM, Fransen HP, Verkaik-Kloosterman J, Buurma-Rethans EJM, Ocké MC: *Dutch National Food Consumption Survey 2007–2010: Diet of Children and Adults Aged 7 to 69 years.* Bilthoven: National Institute for Public Health and the Environment; 2011. RIVM-Report 350050006/2011.
3. **A healthy lifestyle.** [http://www.euro.who.int/en/what-we-do/health-topics/disease-prevention/nutrition/a-healthy-lifestyle]
4. World Health Organization: *Diet, Nutrition and the Prevention of Chronic diseases*, Report of a Joint WHO/FAO Expert Consultation. Geneva: World Health Organization; 2003. WHO Technical Report Series, No. 916.
5. U.S. Department of Agriculture, U.S Department of Health and Human Services: *Dietary guidelines for Americans.* Washington, DC: U.S. Government Printing Office; 2010.
6. Elmadfa I, Kornsteiner M: **Dietary fat intake – a global perspective.** *Ann Nutr Metab* 2009, **54:**8–14.
7. FAO: *FAO Statistical Yearbook.* Rome: Food and agriculture organization of the United Nations; 2004.
8. **Richtlijnen voedselkeuze (Guidelines food choice).** [http://www.voedingscentrum.nl/Assets/Uploads/Documents/Voedingscentrum/Actueel/00_Richtlijnen%20voedselkeuze%202011.pdf]
9. **Hoeveel mag ik per dag eten? (How much should I eat per day?).** [http://www.voedingscentrum.nl/nl/schijf-van-vijf/eet-niet-teveel-en-beweeg/hoe-eet-ik-niet-te-veel.aspx]
10. Ball K, Crawford D, Mishra G: **Socio-economic inequalities in women's fruit and vegetable intakes: a multilevel study of individual, social and environmental mediators.** *Public Health Nutr* 2006, **9:**623–630.
11. Inglis V, Ball K, Crawford D: **Socioeconomic variations in women's diets: what is the role of perceptions of the local food environment?** *J Epidemiol Community Health* 2008, **62:**191–197.
12. Konttinen H, Sarlio-Lähteenkorva S, Silventoinen K, Männistö S, Haukkala A: **Socio-economic disparities in the consumption of vegetables, fruit and energy-dense foods: the role of motive priorities.** *Public Health Nutr* 2012, **16:**873–882.
13. Aggarwal A, Monsivais P, Cook AJ, Drewnowski A: **Does diet cost mediate the relation between socioeconomic position and diet quality?** *Eur J Clin Nutr* 2011, **65:**1059–1066.
14. James WPT, Nelson M, Ralph A, Leather S: **Socioeconomic determinants of health: the contribution of nutrition to inequalities in health.** *BMJ* 1997, **314:**1545–1549.
15. Kroeze W, Werkman A, Brug J: **A systematic review of randomized trials on the effectiveness of computer-tailored education on physical activity and dietary behaviors.** *Ann Beh Med* 2006, **31:**205–223.
16. De Vries H, Brug J: **Computer-tailored interventions motivating people to adopt health promoting behaviours: introduction to a new approach.** *Patient Educ Couns* 1999, **36:**99–105.
17. Kreuter M, Farrell D, Olevitch L, Brennan L: *Tailoring Health Messages. Customizing communication with computer technology.* Mahwah, New Jersey: Lawrence Erlbaum Associates; 2000.
18. Krebs P, Prochaska JO, Rossi JS: **A meta-analysis of computer-tailored interventions for health behavior change.** *Prev Med* 2010, **51:**214–221.
19. Neville LM, O'Hara B, Milat AJ: **Computer-tailored dietary behaviour change interventions: a systematic review.** *Health Educ Res* 2009, **24:**699–720.
20. Brug J, van Assema P: **Differences in use and impact of computer-tailored dietary fat-feedback according to stage of change and education.** *Appetite* 2000, **34:**285–293.
21. **ICT gebruik van personen naar persoonskenmerken (ICT use by person characteristics).** [http://statline.cbs.nl/StatWeb/publication/?DM=SLNL&PA=71098ned&D1=33-133&D2=0-2&D3=a&VW=T]
22. van Deursen AJAM, van Dijk JAGM: *Trendrapport Internetgebruik 2012. Een Nederlands en Europees Perspectief (Trend Report Internet Use 2012. A Dutch and European Perspective).* Enschede: University of Twente; 2012.
23. Sheeran P: **Intention-behavior relations: a conceptual and empirical review.** *Eur Rev Soc Psychol* 2002, **12:**1–36.
24. Abraham C, Sheeran P, Johnston M: **From health beliefs to self-regulation: theoretical advances in the psychology of action control.** *Psychol Health* 1998, **13:**569–591.
25. Contento IR: **Nutrition education: linking research, theory and practice.** *Asia Pac J Clin Nutr* 2008, **17:**176–179.
26. Maes S, Karoly P: **Self-regulation assessment and intervention in physical health and illness: a review.** *Appl Psychol Int Rev* 2005, **54:**267–299.
27. Bandura A: *Social Foundations of Thought and Action: A Social Cognitive Theory.* Prentice Hall: Englewood Cliffs; 1986.

28. Kremers SPJ, de Bruijn GJ, Visscher TLS, van Mechelen W, de Vries NK, Brug J: **Environmental influences on energy balance-related behaviors: a dual-process view.** *Int J Behav Nutr Phys Act* 2006, **3**:9.
29. Brug J, van Lenthe FJ: *Environmental Determinants and Interventions For Physical Activity, Nutrition And Smoking: A Review.* Erasmus MC: Rotterdam; 2005.
30. Abraham C, Michie S: **A taxonomy of behavior change techniques used in interventions.** *Health Psychol* 2008, **27**:379–387.
31. Bartholomew LK, Parcel GS, Kok G, Gottlieb NH, Fernández ME: *Planning Health Promotion Programs: An Intervention Mapping Approach.* San Francisco: Jossey-Bass; 2011.
32. Ovaskainen M-L, Reinivuo H, Tapanainen H, Hannila M-L, Korhonen T, Pakkala H: **Snacks as an element of energy intake and food consumption.** *Eur J Clin Nutr* 2006, **60**:494–501.
33. Karoly P: **Mechanisms of self-regulation: a systems view.** *Annu Rev Psychol* 1993, **44**:23–52.
34. Maes S, Gebhardt W: **Self-regulation and health behavior: the health behavior goal model.** In *Handbook of Self-Regulation.* Edited by Boekaerts PRPM, Zeidner M. San Diego: Academic; 2000:343–368.
35. Kok G, van Empelen P: **Interventie ontwikkeling (Intervention development).** In *Gezondheidsvoorlichting en Gedragsverandering: Een Planmatige Aanpak (Health Education and Behavioral Change: A Systematic Approach).* Edited by Brug J, van Assema P, Lechner L. Assen: Van Gorcum; 2012:125–144.
36. Weinstein ND, Sandman PM, Blalock SJ: **The precaution adoption process model.** In *Health Behavior and Health Education: Theory, Research and Practice.* Edited by Glanz K, Rimer BK, Viswanath K. San Francisco: Jossey-Bass; 2008.
37. Ajzen I: **The theory of planned behavior.** *Organ Behav Hum Decis Process* 1991, **50**:179–211.
38. Lechner L, Kremers S, Meerten R, de Vries H: **Determinanten van gedrag (Determinants of behaviour).** In *Gezondheidsvoorlichting en Gedragsverandering: Een Planmatige Aanpak (Health Education and Behavioral Change: A Systematic Approach). Volume 8.* Edited by Brug J, van Assema P, Lechner L. Assen: Van Gorcum; 2012.
39. Godin G, Kok G: **The theory of planned behavior: a review of its application to health-related behaviors.** *Am J Health Promot* 1996, **11**:87–98.
40. Giskes K, van Lenthe FJ, Brug J, Mackenbach JP, Turrell G: **Socioeconomic inequalities in food purchasing: the contribution of respondent-perceived and actual (objectively measured) price and availability of foods.** *Prev Med* 2007, **45**:41–48.
41. Shaikh AR, Yaroch AL, Nebeling L, Yeh M-C, Resnicow K: **Psychosocial predictors of fruit and vegetable consumption in adults: a review of the literature.** *Am J Prev Med* 2008, **34**:535–543.
42. Guillaumie L, Godin G, Vézina-Im L-A: **Psychosocial determinants of fruit and vegetable intake in adult population: a systematic review.** *Int J Behav Nutr Phys Act* 2010, **7**:12.
43. Anderson ES, Winett RA, Wojcik JR: **Self-regulation, self-efficacy, outcome expectations, and social support: social cognitive theory and nutrition behavior.** *Ann Behav Med* 2007, **34**:304–312.
44. Bogers RP, Brug J, van Assema P, Dagnelie PC: **Explaining fruit and vegetable consumption: the theory of planned behaviour and misconception of personal intake levels.** *Appetite* 2004, **42**:157–166.
45. Brug J, de Vet E, de Nooijer J, Verplanken B: **Predicting fruit consumption: cognitions, intention, and habits.** *J Nutr Educ Behav* 2006, **38**:73–81.
46. Wiedemann AU, Lippke S, Reuter T, Ziegelmann JP, Schwarzer R: **How planning facilitates behaviour change: additive and interactive effects of a randomized controlled trial.** *Eur J Soc Psychol* 2011, **41**:42–51.
47. Kamphuis CBM, van Lenthe FJ, Giskes K, Brug J, Mackenbach JP: **Perceived environmental determinants of physical activity and fruit and vegetable consumption among high and low socioeconomic groups in the Netherlands.** *Health Place* 2007, **13**:493–503.
48. Brug J, Debie S, van Assema P, Weijts W: **Psychosocial determinants of fruit and vegetable consumption among adults: results of focus group interviews.** *Food Qual Prefer* 1995, **6**:99–107.
49. Giskes K, van Lenthe FJ, Kamphuis CBM, Huisman M, Brug J, Mackenbach JP: **Household and food shopping environments: do they play a role in socioeconomic inequelities in fruit and vegetable consumption? A multilevel study among Dutch adults.** *J Epidemiol Community Health* 2009, **63**:113–120.
50. Bere E, van Lenthe FJ, Klepp K-I, Brug J: **Why do parents' education level and income affect the amount of fruits and vegetables adolescents eat?** *Eur J Public Health* 2008, **18**:611–615.
51. De Bruijn G-J, Kremers SPJ, Schaalma H, van Mechelen W, Brug J: **Determinants of adolescent bicycle use for transportation and snacking behavior.** *Prev Med* 2005, **40**:658–667.
52. McCrory MA, Suen SMM, Roberts SB: **Biobehavioral influences on energy intake and adult weight gain.** *J Nutr* 2002, **132**:3830S–3834S.
53. Stafleu A, de Graaf C, van Staveren WA, Schroots JJF: **A review of selected studies assessing social-psychological determinants of fat and cholesterol intake.** *Food Qual Prefer* 1991, **3**:183–200.
54. Inglis V, Ball K, Crawford D: **Why do women of low socioeconomic status have poorer dietary behaviours than women of higher socioeconomic status? A qualitative exploration.** *Appetite* 2005, **45**:334–343.
55. Prochaska JO, Velicer WF: **The transtheoretical model of health behavior change.** *Am J Health Promot* 1997, **12**:38–48.
56. Kok G, Lechner L, Meertens R, Brug J: **Theorieën en methodieken van verandering (Theories and methodologies of change).** In *Gezondheidsvoorlichting en Gedragsverandering: Een Planmatige Aanpak (Health Education and Behavioral Change: A Systematic Approach). Volume 8.* Edited by Brug J, van Assema P, Lechner L. Assen: Van Gorcum; 2012.
57. Strecher VJ, Seijts GH, Kok GJ, Latham GP, Glasgow R, DeVellis B, Meertens RM, Bulger DW: **Goal setting as a strategy for health behavior change.** *Health Educ Behav* 1995, **22**:190–200.
58. Gollwitzer PM, Sheeran P: **Implementation intentions and goal achievement: a meta-analysis of effects and processes.** *Adv Exp Soc Psychol* 2006, **38**:69–119.
59. Shilts MK, Horowitz M, Townsend MS: **Goal setting as a strategy for dietary and physical activity behavior change: a review of the literature.** *Am J Health Promot* 2004, **19**:81–93.
60. Cullen KW, Baranowski R, Smith SP: **Using goal setting as a strategy for dietary behavior change.** *J Am Diet Assoc* 2001, **101**:562–566.
61. Latham GP, Locke EA: **Self-regulation through goal setting.** *Organ Behav Hum Decis Process* 1991, **50**:212–247.
62. Locke EA, Latham GP: **New directions in goal-setting theory.** *Curr Dir Psychol Sci* 2006, **15**:265–268.
63. Mento AJ, Steel RP, Karren RJ: **A meta-analytic study of the effects of goal setting on task performance: 1966–1984.** *Organ Behav Hum Decis Process* 1987, **39**:52–83.
64. Neubert MJ: **The value of feedback and goal setting over goal setting alone and potential moderators of this effect: a meta-analysis.** *Hum Perform* 1998, **11**:321–335.
65. Sniehotta FF, Schwarzer R, Scholz U, Schüz B: **Action planning and coping planning for long-term lifestyle change: theory and assessment.** *Eur J Soc Psychol* 2005, **35**:565–576.
66. Oenema A, Tan F, Brug J: **Short-term efficacy of a web-based computer-tailored nutrition intervention: main effects and mediators.** *Ann Behav Med* 2005, **29**:54–63.
67. Kroeze W, Oenema A, Campbell M, Brug J: **The efficacy of web-based and print-delivered computer-tailored interventions to reduce fat intake: results of a randomized, controlled trial.** *J Nutr Educ Behav* 2008, **40**:226–236.
68. MacKinnon DP, Lockwood CM, Hoffman JM, West SG, Sheets V: **A comparison of methods to test mediation and other intervening variable effects.** *Psychol Methods* 2002, **7**:83–104.
69. van Stralen MM, Kok G, de Vries H, Mudde AN, Bolman C, Lechner L: **The Active plus protocol: systematic development of two theory- and evidence-based tailored physical activity interventions for the over-fifties.** *BMC Public Health* 2008, **8**:399.
70. Prins RG, van Empelen P, Beenackers MA, Brug J, Oenema A: **Systematic development of the YouRAction program, a computer-tailored physical activity promotion intervention for Dutch adolescents, targeting personal motivations and environmental opportunities.** *BMC Public Health* 2010, **10**:474.
71. van Genugten L: **Prevention of weight gain among overweight adults: development and evaluation of a computer-tailored self-regulation intervention.** PhD thesis. Erasmus University, Department of Public Health; 2011.

doi:10.1186/1471-2458-14-47
Cite this article as: Springvloet *et al.*: Planned development and evaluation protocol of two versions of a web-based computer-tailored nutrition education intervention aimed at adults, including cognitive and environmental feedback. *BMC Public Health* 2014 **14**:47.
